# Implementation of the hybrid course on basic wheelchair service provision for Colombian wheelchair service providers

**DOI:** 10.1371/journal.pone.0204769

**Published:** 2018-10-04

**Authors:** Yohali Burrola-Mendez, Maria Luisa Toro-Hernández, Mary Goldberg, Jon Pearlman

**Affiliations:** 1 Department of Rehabilitation Science and Technology, University of Pittsburgh, Pittsburgh, Pennsylvania, United States of America; 2 International Society of Wheelchair Professionals (ISWP), University of Pittsburgh, Pittsburgh, Pennsylvania, United States of America; 3 Consejo Nacional de Ciencia y Tecnología, México City, México; 4 School of Physical Therapy, Universidad CES, Medellín, Colombia; Universita degli Studi di Perugia, ITALY

## Abstract

**Introduction:**

People with mobility impairments often rely on wheelchairs as their primary means of mobility. Untrained wheelchair service providers may provide inappropriate wheelchairs and services which result in negative consequences in wheelchair users’ health, quality of life, safety, and social participation. This study aimed to evaluate the influence of the Spanish Hybrid Course on Basic Wheelchair Service Provision, a training based on the World Health Organization’s Wheelchair Service Training Program-Basic Level, to increase knowledge in basic level wheelchair service provision among a group of wheelchair service providers from Colombia. In addition, we developed a satisfaction survey which participants completed after the training to understand levels of satisfaction with the training intervention.

**Methods:**

A quasi-experimental study was conducted to evaluate changes in basic level wheelchair knowledge using the Wheelchair Service Provision–Basic Test. Paired sample *t* tests were used to assess pre-and post-training changes in basic level wheelchair knowledge. The Hybrid Satisfaction Survey was developed in collaboration with a multidisciplinary, international stakeholders’ group. The survey’s construct of interest was level of satisfaction determined by interaction, instructor, instruction methodology, content, and technology, using a five-point Likert scale (0 = strongly disagree to 4 = strongly agree). The survey was completed anonymously after the training intervention and analyzed using frequencies and percentages.

**Results:**

Fifteen wheelchair service providers in Colombia completed the Spanish Hybrid Course. Mean post-scores were significantly higher (Mean (M) = 56.13, Standard Deviation (SD) = 7.8), than pre-assessment scores (M = 50.07, SD = 8.38, t(14) = 4.923, p = <0.0001). Participants who completed the surveys (N = 15) reported that the Spanish Hybrid Course was well received, with 98.66% of responses distributed in favorable levels (>3).

**Conclusions:**

The Spanish Hybrid Course proved to be effective in increasing basic level wheelchair knowledge with a high satisfaction level among participants. Further testing is needed to evaluate the effectiveness of this course across different individuals and countries as a potential tool to build professional capacity in basic level wheelchair provision.

## Introduction

The World Bank estimates that one billion people, or 15% of the world’s population, experience some form of disability, and its prevalence is higher in low- and middle-income countries (LMICs) [1]. In Latin America, 12.4% of the population, approximately 73 million people, live with at least one type of disability [2, 3]. Disability is more prevalent among women and the most economically and socially vulnerable groups: Lower-income persons, older persons, indigenous people, Afro-descendants, and inhabitants of rural areas [2]. In fact, it is estimated that around 80% of people with disabilities in Latin America live in poverty, and that mobility and vision impairments are the most common types of disability [2, 4, 5]. The World Bank classification by gross national income reports in 2018 indicate that Latin America is comprised of 4 lower-middle-income, 12 upper-middle-income, and 4 high-income countries [6, 7]. Spanish is the official language in 80% of the countries within the region and is the second most spoken language worldwide [8, 9]. The United Nations Convention on the Rights of People with Disabilities (UNCRPD), which promotes effective human rights for all persons with disabilities, has been signed and ratified by 95% of countries in Latin America [10–12]. Despite the advances in the legal recognition of the rights of people with disabilities, the situation in Latin America continues to be characterized by great inequality, socioeconomic gaps, and social vulnerability [13].

People with mobility impairments often rely on the use of assistive technology devices, such as wheelchairs, as their primary means of mobility. An appropriate wheelchair can prevent secondary health complications, enhance quality of life, improve safety, and facilitate access to human rights such as education, healthcare, and employment [14–19]. Article 20 of the UNCRPD emphasizes the need to promote personal mobility with the greatest independence by providing training to persons with disabilities and personnel providing services to them [20]. In addition, Articles 4, 9, 25, 26, and 32 mandate that an appropriate wheelchair is delivered by trained personnel [10]. Nevertheless, evidence highlights a shortage of education and training in wheelchair service provision available to personnel globally [21, 22]. Inadequate education and training of wheelchair service providers can result in inappropriate wheelchair provision, which has negative impacts in quality of life, health, safety, and other basic human rights [14–20]. This situation may be more consequential in LMICs where the incidence of disability is higher, there is a limited range of quality affordable wheelchairs, disability and poverty operate in a cycle, and people with disability often are marginalized [2, 21, 23, 24].

The World Health Organization (WHO), with the support of the United States Agency of International Development (USAID), has published a series of Wheelchair Service Training Packages (WHO WSTP) to assist nations in fulfilling the UNCRPD mandate of supporting providers’ training and helping to bridge the gap in training disparity across less-resourced settings [14, 25]. To date, there are five WHO WSTPs: Basic, intermediate, managers’, stakeholders’, and trainers’ packages [25–29]. The WHO has officially translated these packages into different languages, including Spanish, and offers access to the course materials free of charge from the WHO website [25, 30]. Since the adoption of the UNCRPD in 2006, there is a growing commitment in Latin America to provide educational programs and training in wheelchair service provision locally that enhance positive effects in the community. One such initiative, the Latin American Seating Symposium, has been held every two years as a regional effort to promote access, standard of practice, and effective use of assistive technology devices [31]. In the symposia, internationally recognized continuing education programs like the Wheelchair Skills Program and the WHO WSTPs are delivered as workshops [27, 28, 32]; however, there is still an overwhelming need for training and appropriate services that is not being met by the current workforce.

With the purpose of offering alternative learning methodologies that could be scaled across countries and regions for global capacity building, in 2016, the International Society of Wheelchair Professionals (ISWP) developed a Hybrid Course in English and Spanish using the official versions of the WHO WSTP-Basic (WHO WSTP-B) in both languages [30, 33]. The Hybrid Course consists of online modules designed for low-bandwidth internet access that reduce the in-person training exposure to 3 days, making it less expensive and easier to scale-up [33]. The English Hybrid Course proved to be effective in increasing basic level wheelchair service provision knowledge in a group of graduate students from Rehabilitation Sciences, based on the scores of the validated ISWP Wheelchair Service Provision-Basic Test [33, 34].

While initial learning outcome results offered some evidence that the Hybrid Course may be an effective mechanism to train wheelchair service provision content, the course was tested in English exclusively in a high-resource environment and it did not capture satisfaction among participants [33]. We needed to evaluate the Spanish Hybrid Course in different contexts and explore trainees’ satisfaction level post-training to determine if it is an effective and well-received learning method to build capacity for wheelchair service providers in Latin America. Thus, the purpose of this exploratory study was to test the Spanish Hybrid Course in Colombia. This country was selected for this first training intervention due to the presence of local facilitators who previously were trained through the WHO WSTP-B and the WHO WSTP–Trainer’s package, as well as the research team’s local partnerships and access to key stakeholders.

The specific aims of this study were to: (1) develop a satisfaction survey; then (2) evaluate the effect of the Spanish Hybrid Course among a group of wheelchair service providers located in Colombia. We hypothesized that trainees would have higher scores on the ISWP Wheelchair Service Provision-Basic Test after receiving the training, and that they would report overall high levels of satisfaction with the training. This study would provide further understanding of the Spanish Hybrid Course to help determine its potential implementation in other countries.

## Methods

Each specific aim was completed in sequence; the methods are described below.

### Specific Aim 1: Development of the satisfaction survey

For this specific aim, we followed the methodology implemented for the development of the Hybrid Course [33] in which the same international and multidisciplinary group of stakeholders oversaw and guided the development of the Hybrid Satisfaction Survey. This group, the Hybrid Subcommittee (HSC), was comprised of 8 members from lower-middle to high-income economies (Brazil, Canada, Colombia, India, Mexico, Philippines, United Kingdom, and the United States of America) with experience delivering and developing wheelchair training programs in different settings [33].

The authors of this manuscript and the HSC developed the Hybrid Satisfaction Survey to evaluate levels of satisfaction among participants after the Hybrid Course training intervention. The construct of interest was levels of satisfaction after training experiences determined by 5 sub-domains: Interaction, instructor, instruction methodology, content, and technology. The items were selected from existing surveys that evaluated satisfaction with online learning [35], satisfaction with blended learning in a gender-segregated environment [36], and satisfaction with blended learning [37]. The selected items were adapted to reflect the content of the course. The items’ adaptation followed best practices for item construction that included: Using plain language, avoiding double-barreled questions, and writing in a positively worded direction. [38, 39]. The survey used a five-point Likert scale (4 = strongly agree, 3 = agree, 2 = neither agree nor disagree, 1 = disagree, 0 = strongly disagree) that prompted participants to indicate the degree to which they agreed with each statement in the sub-domains. An open-ended question was included at the end of each sub-domain to encourage participants to provide suggestions and feedback. In addition, the sub-domain “Content” had a multiple-choice question that was analyzed individually: “For the course that you completed, would you prefer: a) to have more online modules, b) to have more in-person practice, c) to have more online materials and in-person practice, d) nothing to change, or e) other (entry box).” The Hybrid Satisfaction Survey was hosted online in Qualtrics®. Since the lead author and 3 HSC members were bilingual in English and Spanish, the Hybrid Satisfaction Survey was developed simultaneously in both languages and can be accessed in the Supporting Information section of the article (S1 File).

### Specific Aim 2: Implement the hybrid course with a group of wheelchair service providers from Colombia

#### Study design

This study was designed as a pre/post repeated measures study conducted to evaluate changes in basic level wheelchair knowledge and a post-assessment design to evaluate levels of satisfaction after the implementation of the Spanish Hybrid Course in a group of wheelchair service providers in Medellin, Colombia. The study was approved by the University of Pittsburgh Institutional Review Board. The study reporting complied with the Template for Intervention Description and Replication (TIDieR) checklist for reporting of interventions [40].

#### Study sample

The sample was selected using a purposive sampling method guided by the local stakeholders’ input. Universidad CES in Medellin, Colombia, led the recruitment, enrollment, and delivered the Hybrid Course. The inclusion criteria included individuals who: 1) worked locally in wheelchair service delivery; and 2) who had not taken the ISWP Wheelchair Service Provision–Basic Test. Participants who were simultaneously participating in another wheelchair-related study were excluded.

The training was provided at no cost to participants. Therefore, to reduce attrition, Universidad CES sent a call for applications to disability and rehabilitation-related stakeholders (hospitals, public assistive technology, physical therapy programs, local and international non-governmental organizations, and community leaders, among others). Participants’ applications included their supervisor’s approval and a letter of commitment to complete online and in-person training activities. Universidad CES selected 15 out of the 18 applications received based on their work experience and potential to apply and to disseminate the content. The number of participants for the course was selected based on the size of the course and trainer-trainee ratio that is suggested by WHO to foster an appropriate learning environment since the program had a significant amount of hands-on practical sessions [41].

#### Intervention

Table 1 presents the study overview and timeline, and Table 2 provides the TIDieR checklist for reporting interventions. Three local facilitators worked interprofessionally during the planning phase (recruiting trainees, identifying wheelchair recipients and procuring wheelchairs) and the training delivery. They were a physical therapist, a biomedical engineer, and a physical medicine and rehabilitation doctor. The first two participated in a training of trainers course specific to the WHO WSTP [42]. The physiatrist had more than 10 years of experience in wheelchair provision and also received training as a trainer in the International Classification of Functioning, Disability, and Health [43].

**Table 1:** Study overview and timeline.

Activity	Days	June—September 2016						October 2016				November 2016			
								1	2	3	4	1	2	3	4
**Recruitment**	90														
**Training Intervention**	42														
• Pre-Assessments	9														
• Online training	15														
Phase 1. Modules 1–4	8														
*Recitation*															
Phase 2. Modules 5–8	7														
*Recitation*															
• In-person training	3.5														
• Post-Assessments	8														

**Table 2:** TIDieR checklist for reporting interventions.

**Intervention name**	International Society of Wheelchair Professionals (ISWP) Hybrid Course on Wheelchair Service Training Package—Basic Level/Spanish version	
**Why:**	There is a need to train wheelchair service providers in a more flexible and scalable manner. This program has never been tested in Spanish.	
**What**	*Materials*	Adobe Connect, Internet access, one big accessible classroom, assessment beds/mats, demo wheelchairs, donated wheelchairs, foam, participants' handbooks, participants' workbooks, trainers' manuals, training program evaluation forms, posters, chairs, wheelchairs forms, one whiteboard, computer, projector, cushion fabrication toolkits, home maintenance toolkit. The list of training resources, materials, and tools is included in the WHO Wheelchair Service Training Package Trainer's Manual Basic Level [41].
	*Procedures*	Participants had one week prior to the beginning of the course to complete pre-assessments. The ISWP Hybrid Course consisted of two weeks of asynchronous online training with two synchronous online meetings (recitations). The online training followed 3.5 days of in-person training. After this, participants had one-week to complete the post-assessments.
**Who provided**	*Online section*:	ISWP staff and ISWP Hybrid Course developer
	*In-person section*:	Three trained instructors (Physical Therapist, Medical Doctor, and Biomedical Engineer)
**Models of delivery**	One-group of 15 participants took the ISWP Hybrid Course simultaneously and asynchronously. During recitations, participants interacted with each other and with the instructors. In the in-person sessions, participants practiced with wheelchair users in groups of three.	
**Where**	The in-person sessions were facilitated in one classroom of 50 square meter at Universidad CES in Medellin, Colombia.	
**When and how much**	*Pre-assessment*:	The ISWP Wheelchair Service Provision–Basic Test, online test, which takes approximately one hour to be completed
	*Online section*:	Two weeks, 8 online modules, 2 synchronous recitations of 90 minutes each.
	*In-person section*:	Three and a half days, 8 hours per day.
	*Post—assessments*:	The ISWP Wheelchair Service Provision–Basic Test, online test, approximately one hour to complete. The ISWP Hybrid Course, online, approximately 30 minutes to complete.
**Tailoring:**	The demo wheelchairs used were from local context.	
**Modifications:**	None	
**How well**	*Fidelity*:	Not tested
	*Adherence*:	Overall, the intervention was delivered as planned. Only one wheelchair user did not participate in the training. Participants had to work in larger groups. There was not a machine shop at Universidad CES and one wheelchair needed to be adjusted. The adjustment was made at a later appointment with one of the instructors and the wheelchair supplier.

#### Online training

The online learning was divided into two sequential phases (Table 1). At each phase, participants reviewed the content and completed the required activities asynchronously to meet the learning outcomes (S2 File). The description of the online modules’ content, course activities, instructional materials used and learners’ interactions have been described elsewhere [33]. After the completion of each phase, an online meeting (recitation) was organized synchronously between participants and trainers. During the recitations, trainers reinforced the key points of the modules, answered questions, discussed topics, and promoted interaction among participants. Recitations lasted 90 minutes, were recorded, and made available to participants and trainers. ISWP staff attended the recitations to help coordinate the sessions and provide support, if needed.

#### In-person training

After the completion of the online portion of the course, participants attended 3.5 days of in-person training led by local trainers at Universidad CES in Medellin, Colombia (Table 1 and S2 File). Ten wheelchair users who previously were assessed for a wheelchair by trainers were invited to participate as volunteers for the in-person sessions. Additionally, 5 wheelchair users who had received a new wheelchair in the past year were invited as volunteers for the follow-up practical session (Day 4, Practical 3 in S2 File). The goal was for three participants to work with one wheelchair user volunteer during each practical session. On the last day, wheelchair users received the benefit of a new appropriate wheelchair provided by the trainees.

#### Outcome measurements

A priori, we defined that our primary measure of interest was knowledge change, and our secondary measure, participants’ level of satisfaction. We deemed these two outcomes relevant to evaluate the influence of the Spanish Hybrid Course and to plan for a potential larger future study.

To evaluate knowledge change, participants completed the ISWP Wheelchair Service Provision–Basic Test one week before and after the training intervention. The aforementioned test has shown validity evidence for measuring basic level wheelchair service provision knowledge independent of geographic location [34]. The test consisted of 19 sociodemographic questions and 75 multiple-choice questions that evaluated basic wheelchair service delivery. The sociodemographic questions included general characteristics of the test takers such as age, gender, educational level, profession, employment status, years of experience in wheelchair service provision, work setting, and motivation to take the training. Some demographic questions, such as work setting, age group served, and motivation to take the training allowed multiple answers, and participants were asked to select all applicable options. The 75 multiple-choice questions covered seven domains of wheelchair service delivery: 1) assessment, 2) prescription, 3) fitting, 4) production, 5) user training, 6) process, and 7) follow-up and maintenance as described in the WHO WSTP-B [34]. The domains had different weights based on the pre-set number of questions that each domain was allocated. Each domain had a pool of questions created to reduce the likelihood of receiving the same question when taking the test multiple times. In addition, the test settings included: 1) random distribution of questions and answers from each domain’s pool of questions; 2) force completion that required participants to complete the test in one-time entry; and 3) immediate test scores and the ability to review correct and incorrect answers [33, 34] Test scores greater than or equal to 53 points (70% of the total points) were considered passing scores. Participants received an email with instructions on how to log into the testing platform, Test.com®, and contact information for ISWP staff and the local lead trainer in case of technical problems or questions. Participants were instructed to complete the test without accessing course materials. The test was completed in Spanish.

To evaluate levels of satisfaction after the training intervention, participants were invited to complete the Hybrid Satisfaction Survey online anonymously. Participants received an email with the survey link. The survey was hosted in Qualtrics® and included a cover letter with instructions, the purpose of the questionnaire, a confidentiality statement, and contact information for questions or technical issues.

#### Data management and analysis

All data was collected in a Test.com® and Qualtrics® database, exported into a CSV file and then into SPSS® Version 24.0 where all calculations were performed. Data was visually inspected for distributional assumptions using Q-Q plots. Mean values (*M*), Standard Deviation *(SD)*, and confidence intervals of 95% were calculated.

Differences between the pre- and post- test total scores and domain scores were calculated and visually inspected for distributional assumptions using Q-Q plots. Box plots were used to graphically present the pre- and post-test total scores and domain scores using percentages. The total test scores and the domain scores were converted into percentages by dividing the score obtained by the total number of questions (of the test or the specific domain) and then multiplying by 100. The percentages were used to construct the box plots of the pre- and post-test domain scores. For the primary outcome, knowledge change, a paired sample *t* test was calculated when the differences were normally distributed. All analyses were carried out using an alpha level of 0.05.

For the secondary outcome, participants’ satisfaction, survey responses were analyzed using frequencies and percentages. Each question was analyzed individually by summing the number of times the type of response was selected (4 = strongly agree, 3 = agree, 2 = neither agree nor disagree, 1 = disagree, 0 = strongly disagree). Then, the frequencies of the type of response were converted into percentages by dividing each Likert-type scale response by the total number of responses received and then multiplying by 100. Open-ended questions were analyzed individually.

## Results

A total of 15 participants were recruited; all of them completed the pre-and post-assessments, and therefore, there were no dropouts. Summary statistics of participants’ characteristics are described in Table 3. Regarding work settings, outpatient was the most common selection (n = 9), while in-patient was the least frequent selection (n = 3). All participants selected adults as one of the age groups served (n = 15); early childhood and older adults were the second most common selection (n = 9). Professional growth (n = 12) followed by personal growth (n = 7) were the most common motivation to take the trainings.

**Table 3:** Characteristics of the study population.

Characteristic	Hybrid Colombia (n = 15)
Age, mean (SD)	36.80 (10.25)
Sex, Female, n (%)	10 (66.7)
Educational level, n (%)	
<Bachelor	2 (13.3)
Bachelor	11 (73.3)
Graduate degree	2 (13.3)
Profession, n (%)	
Occupational Therapy	1 (6.6)
Physical Therapy	7 (46.6)
Social worker	2 (13.3)
Prosthetic and orthotic technician	1 (6.6)
Biomedical engineer	1 (6.6)
Community leader	1 (6.6)
Physical educator	1 (6.6)
Nurse	1 (6.6)
Employment status, n (%)	
20 hours/week	3 (20.0)
40 hours/week	12 (80.0)
Work setting, n (%)	
Hospital	4 (26.7)
Academic	5 (33.3)
Outpatient	9 (60.0)
In-patient	3 (20.0)
Age group served, n (%)	
early childhood	9 (60.0)
adolescents	8 (53.3)
adults	15 (100)
older adults	9 (60.0)
Wheelchair services provision, Years, n (%)	
Less than 3 years	11 (73.3)
4–7 years	1 (6.7)
8 or more years	3 (20.0)
Previous wheelchair courses, n (%)	6 (40)
Service to wheeled mobility, Hours, n (%)	
Less than 3 hours	4 (26.7)
3–20 hours	8 (53.3)
21 or more hours	3 (20.0)
Motivation training, n (%)	
professional growth	12 (80.0)
personal growth	7 (47.7)
required by academic program	4 (26.7)
Member of an organization*, n (%)	11 (73.3)

SD: Standard deviation

*Organization providing wheelchair services

**Fig 1:**
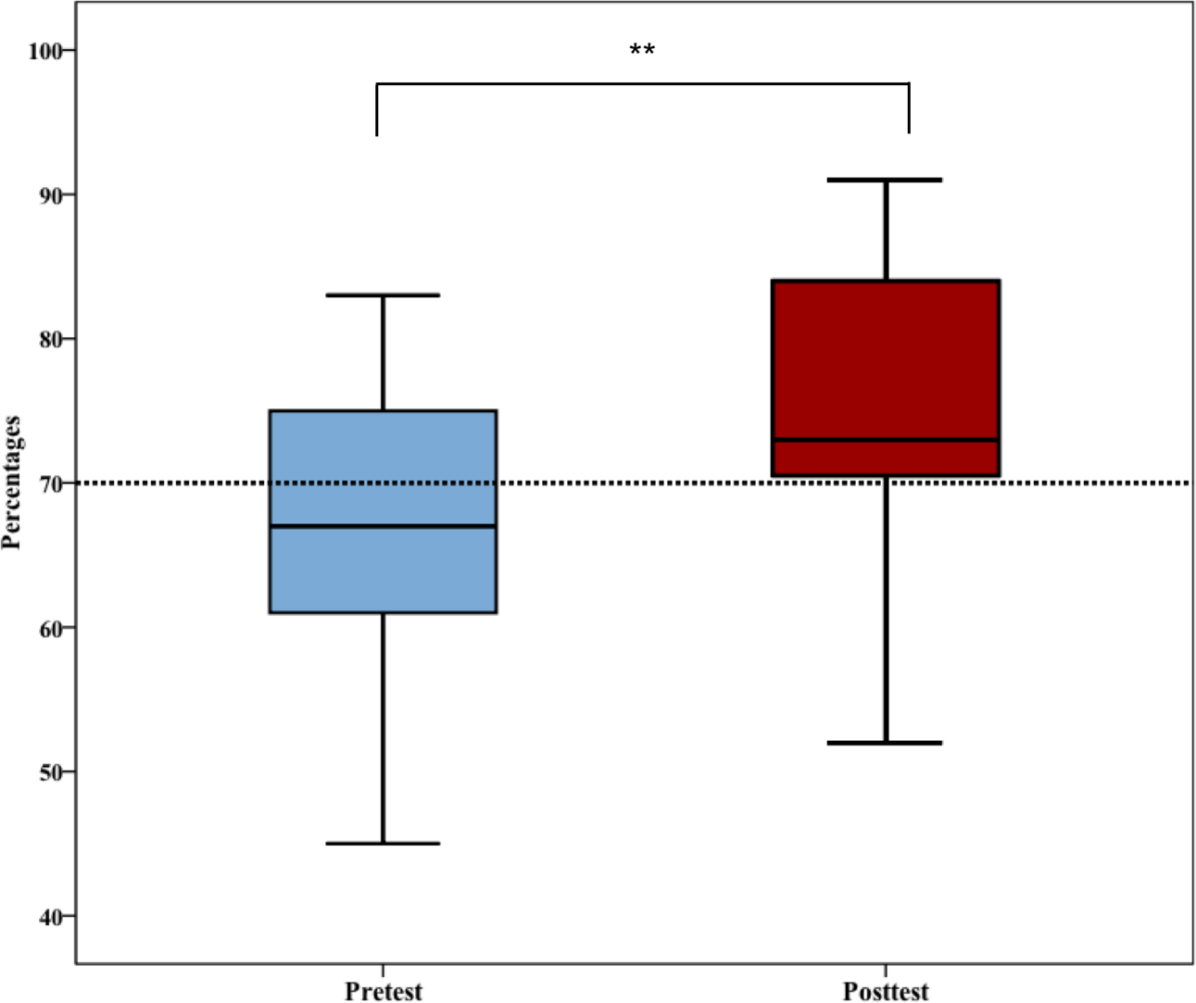
Box plots of pre- and post-total test scores. Comparison of the pre- and post-test total scores. Significant differences in total test scores were observed between groups. **: p<0.0001.

The differences between the pre- and post-test total scores and the pre- and post-domain scores were normally distributed. A paired-sample t-test indicated that post-assessment scores were significantly higher (M = 56.13, SD = 7.8) than pre-assessment scores (M = 50.07, SD = 8.38), t(14) = 4.923, p = <0.0001 (Table 4). Fig 1 presents box plots of pre-and post-test total scores.

**Table 4:** Descriptive statistics and mean changes of pretest and posttest scores, N = 15.

Test (No. Questions)	Pretest N = 15			Posttest N = 15			Mean change from posttest to pretest (95% CI)
	*M (SD)*	Min	Max	*M (SD)*	Min	Max	
Domains							
Assessment (19)	13.93 (2.19)	9	17	16.33 (2.06)	13	19	2.4 (1.38, 3.42) *
Prescription (12)	7.87 (1.85)	5	11	9.13 (1.92)	5	11	1.26 (-0.04, 2.58)
Fitting (10)	4.3 (1.53)	2	7	4.2 (1.78)	0	7	-0.06 (-1.22, 1.08)
Production (5)	2.8 (1.52)	0	5	3.4 (0.91)	2	5	0.60 (-0.05, 1,25)
User's Training (15)	9.33 (2.29)	5	12	10 (2.3)	4	13	0.66 (-0.86, 2.20)
Process (10)	7.13 (2.67)	2	10	8.2 (2.43)	0	10	1.06 (-0.24, 2.38)
Follow up (4)	2.73 (0.703)	2	4	2.87 (1.19)	0	4	0.13 (-0.41, 0.68)
Total scores (75)	50.07 (8.38)	34	62		56.13 (± 7.8)	39	68	6.06 (3.42, 8.71)*

M: Mean

SD: Standard deviation

*paired sample t-test significant at p<0.05 level

**Fig 2:**
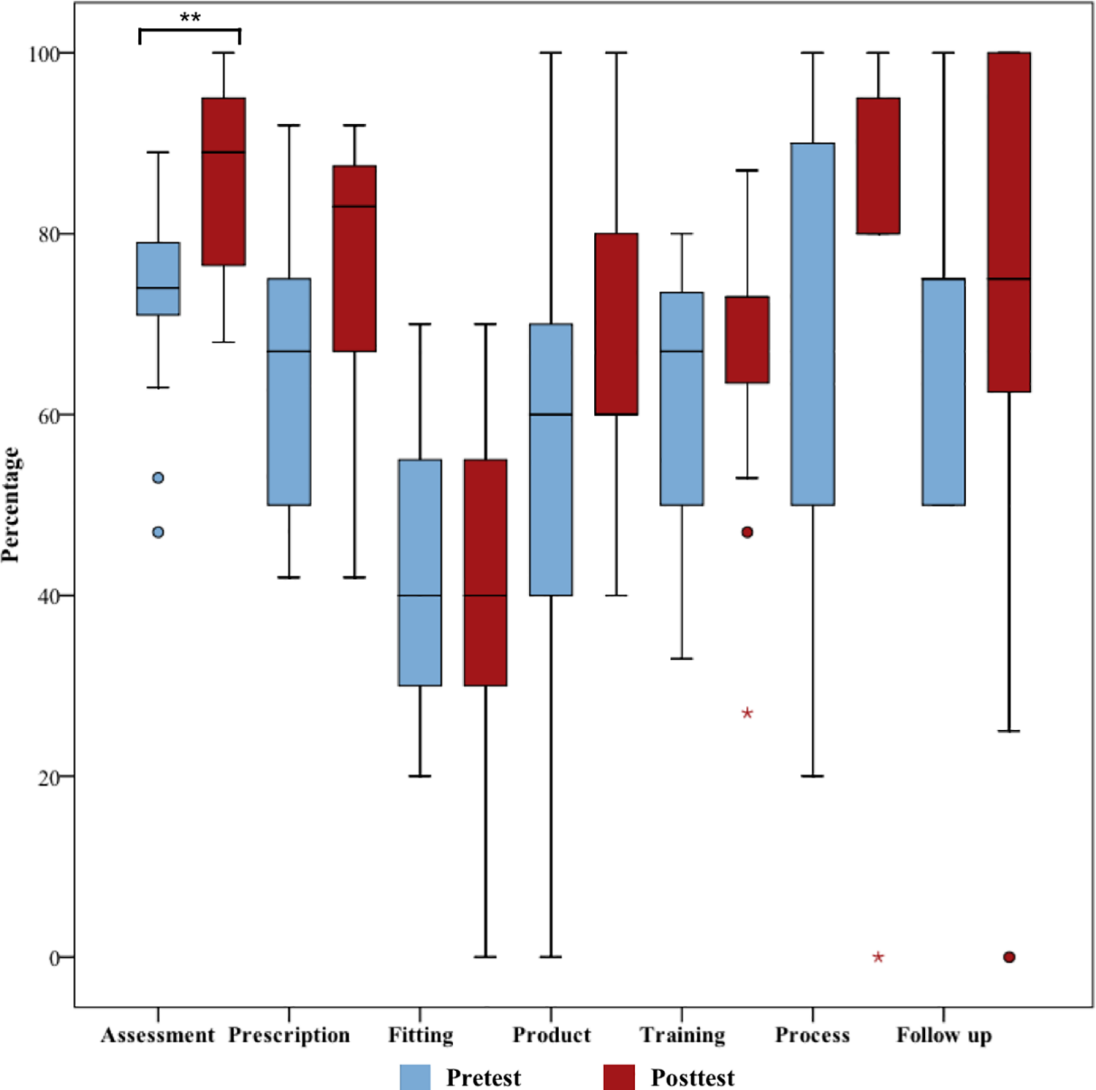
Box plots of pre- and post-domain scores presented in percentages. Comparison of the pre- and post-test total domain scores. Significant differences were observed between pre- and post-test total Assessment scores. The circles represent outliers, values between 1.5 and 3 box lengths, and an asterisk represents extreme outliers, a value more than 3 times the interquartile range. **: p<0.0001.

Knowledge change per domain was explored; all domains, except for “Fitting” presented an increase in mean scores between pre-test and post-test. “Assessment” reported a significant difference in the pre-test scores (M = 13.93, SD = 2.19) and posttest scores (M = 16.33, SD = 2.06),t(14) = 5.041, p = <0.0001 (Table 4). In Fig 2, blox plots of the seven domains were compared between pre-and post-test scores.

**Fig 3:**
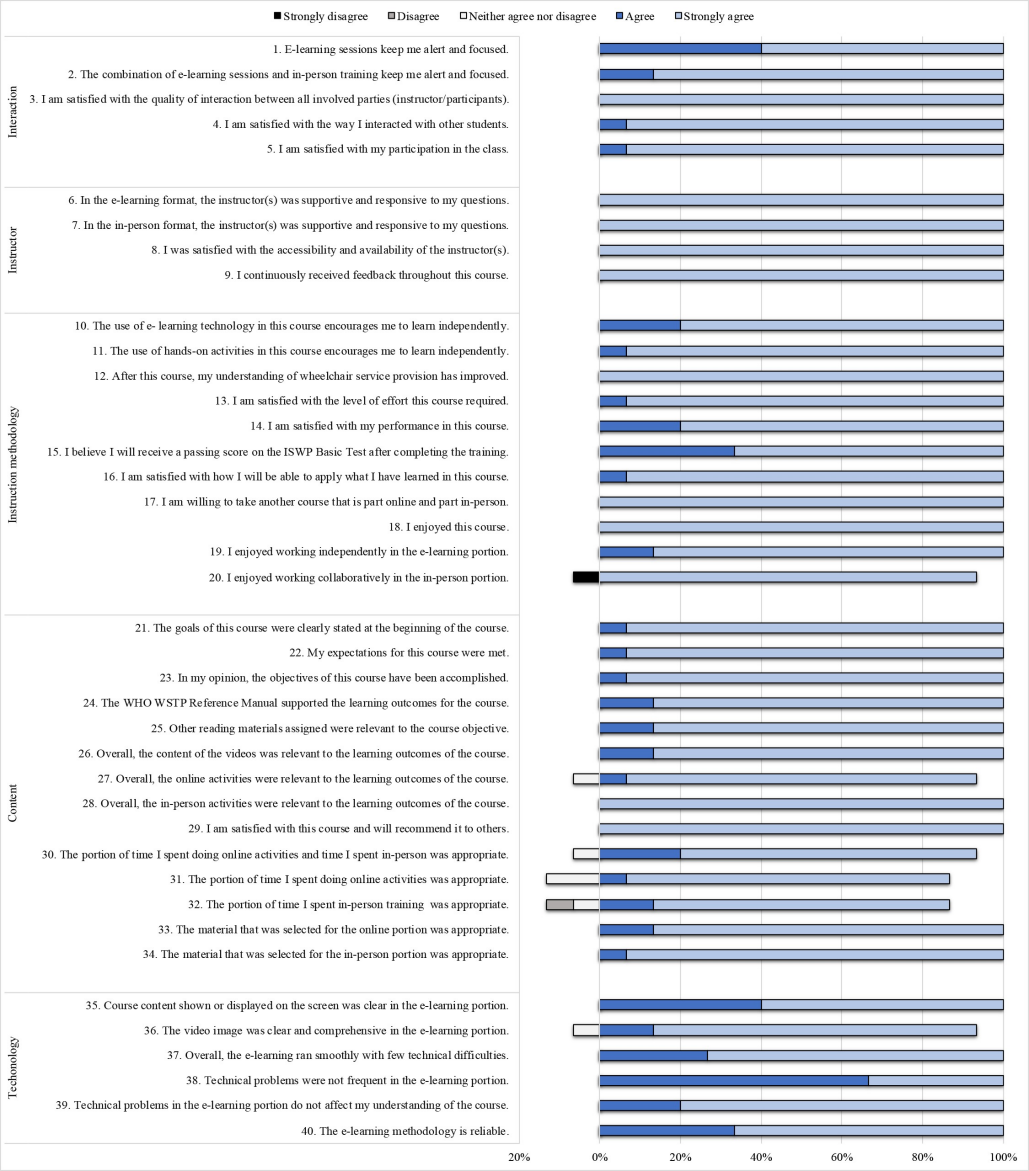
Hybrid satisfaction survey responses in percentages, N = 15.

Fifteen anonymous Hybrid Satisfaction Surveys were received and analyzed. All participants enjoyed the course, were satisfied with the course, were willing to take another hybrid course, would recommend the course, and considered that their understanding of wheelchair service provision improved. Overall, the Spanish Hybrid Course was positively evaluated, reporting 98.66% of the responses distributed in favorable levels (3 = agree and 4 = strongly agree) (Fig 3).

Open-ended questions were individually analyzed; results showed that most students felt highly satisfied with the course (Table 5). Suggestions to improve the online portion included to revise some questions on the quiz and to increase the time allocated to review modules A5-A8. Six participants suggested increasing the in-person time; 2 suggested increasing both the in-person and the online time, and 5 suggested leaving the course as it is.

**Table 5:** Participant’s comments after completing the hybrid training.

Domains	Comments*	
	Positive	Constructive
**Interaction**	Totally satisfied with the work done. Excellent organizers and instructors in the topics discussed. The topics and their duration were appropriate.	
**Instructor**	Everything stated by the instructors was exceptional. They were ready to all the concerns, collaborators, respectful and affordable to all the concerns and proposals of each of the group.	In the online section, some answers were wrong. The time allocated to complete the second online portion (A5-A8) was short.
**Instruction methodology**	I had never taken a course in this format [hybrid]. To be honest, I was afraid. But after taking this course, I am willing to take more courses in this format.	
**Content**	The hybrid methodology is very interesting because it allows me to work individually from my space and in the in-person interact with professionals from different fields and experiences that strengthen my work	I would have liked to have more time in-person so we could help more wheelchair users with this course.
**Technology**	Only satisfaction and gratitude to the organizers, sponsors, the university, the professors … excellent!	

*Comments were translated from Spanish to English

## Discussion

We evaluated the influence of the Spanish Hybrid Course among a group of wheelchair service providers in Medellin, Colombia and developed a satisfaction survey to capture participants’ level of satisfaction after the training intervention. The Spanish Hybrid Course had a statistically significant influence on the total score of the ISWP Wheelchair Service Provision–Basic Test score and received overall high satisfaction ratings, demonstrating the potential value of the Spanish Hybrid Course to train wheelchair service providers at the basic level.

This project demonstrates a successful strategy that could address the lack of trained personnel and promote coordination among professionals who work with people with disabilities. In fact, lack of a coordinated multi-sectoral approach, including a disconnect between different levels of rehabilitation (e.g., acute and community-based rehabilitation), and a shortage of skilled personnel have been identified as barriers to achieving global action on the disability agenda in LMICs [44–47]. The results of this study call for more research to explore training models that can be scaled and cost-effective pathways to building a sustainable training force, including interprofessional training that may contribute to better coordinated services. For instance, a more sustainable partnership to support capacity building and a deeper understanding of global health needs could be explored, as proposed by physiotherapy programs in Ireland and Uganda [48].

Specific to Colombia, the *Disability Law*, enacted in 2013 after the ratification of the UNCRPD, states that the government will warrant access to appropriate assistive technology to promote social inclusion, participation, habilitation, and rehabilitation [49]. Colombia is undergoing a post-armed conflict era that focuses on building a peaceful environment by including vulnerable groups that traditionally have been discriminated against and marginalized, [50] including people with mobility impairments who need a wheelchair [51]. However, the country lacks national wheelchair provision guidelines, including defined competencies that professionals must have to be involved in wheelchair provision. Colombia has two programs specific to the context of wheelchair provision. The *Assistive Technology Banks* in the capital, Bogota, which are meant to provide assistive technology, including wheelchairs, to its poorest residents [52]. The requirement for professionals to work in provision is to be a rehabilitation professional with training or experience in the assistive technology field [53]. The other program is the *Protocol for Assistive Technology Provision*, from the military forces, which requires a prescription from a medical doctor and support of an interdisciplinary rehabilitation team for users to receive a wheelchair [54]. Both programs lack detail on the minimum competencies that professionals must have for wheelchair service provision. An intervention as proposed in this study could be explored to warrant basic wheelchair knowledge of professionals working in the aforementioned programs.

Sixty-seven percent of the pretest scores were below the passing cutoff of the test (53 points), which could indicate the need to update and/or promote training in wheelchair service provision. However, it is important to note that the majority of participants (73%) self-reported less than three years of experience working with wheelchair users. This situation may reflect the limited training time (or lack of training) professional rehabilitation training programs provide in wheelchair service provision [22] and/or the need to integrate training into existing curricula in universities to guarantee graduates are adequately prepared and skilled to assume the wheelchair provision process [21]. Increasing the sample size and including experienced wheelchair providers could help to explore the possible relationship between basic level wheelchair knowledge and wheelchair service provision experience. The Spanish Hybrid Course had a statistically significantly influence on the total ISWP Wheelchair Service Provision-Basic Test group’s mean score, and seventy-four percent of the post-test scores were above the passing cutoff for the test. These findings correlate with our previous study in which we evaluated the effect of the English Hybrid Course in a group of Rehabilitation Science students [33]. Furthermore, mean scores were calculated per domain to explore specific knowledge change. All domains, except "Fitting", reported an increase in post-test scores, an observation that also occurred in our previous evaluation of the Hybrid Course in English [33]. “Assessment” was the only domain which had a statistically significant increase. While the aim of the study was exploratory, and we did not hypothesize statistically significant changes in total test scores and domain scores, we decided to analyze the results by conducting a post-hoc paired-sample *t* test to gather data for future studies. It is important to note that the alpha level for multiple testing was not adjusted because this was an exploratory study with a small sample size, and we were concerned about the potential of making a Type II error [55–57]. A larger sample size, that could be achieved by conducting this training multiple times in different settings to comply with the WHO WSTP suggestion for group size, could help to determine the Spanish and English Hybrid Course's impact on each specific domain and to detect future content modifications, if needed.

A potential strength of this study is that all phases of the research process included stakeholder collaboration. This approach suggests that the study’s results will be more usable, relevant, potentially scalable, and transferable to the community [58–60]. Additionally, the group was comprised of diverse professionals, which supports that interprofessional collaboration has led to improvements in healthcare and must be promoted in educational and healthcare training [61]. Literature specific to the wheelchair field indicates that a team approach of a range of trained professionals and non-professionals is required to develop an appropriate system that increases access to wheelchair technology [14, 21, 25, 62]. Furthermore, adult learning theories indicate that discussions among a diverse group of professionals increase the amount of practical knowledge and the likelihood that individuals within the group are able to learn [63, 64]. In this training, an assistive technology program manager participated, which may have helped to promote the program implementation and advocate for the buy-in of other decision-makers. It may be necessary to investigate further whether having this type of in-depth training reflects changes in current practice. Additionally, having a community leader, who was also a wheelchair user, as a participant, aligns with Colombia’s community-based rehabilitation guidelines that indicate the importance of having community workers close to the health sector so they can support referrals and basic maintenance and repairs, and help the sector understand actual needs of people with disabilities and their families [65]. To improve the reporting of this training intervention and, ultimately, its replicability, we used the TIDier checklist, a guide of 12 items that enhance the quality of description of interventions [40].

Participants reported overall high levels of satisfaction after the training intervention in the five sub-domains of the satisfaction survey: Interaction, instructor, instruction methodology, content, and technology. One element that may be a contributing factor to participants’ satisfaction was that the facilitators had previously been trained in the WHO WSTP-Trainer’s package which takes into consideration important adult learning strategies [42]. In addition, the facilitators were from the local context which suggests that they are more familiar with contextual issues and able to tailor the training [66]. We treated the Likert-style responses as ordinal data because we did not assume that the difference between responses was equidistant even though the numbers assigned to those responses were equal [67]. As often recommended, we used non-parametric tests that included frequencies and percentages of responses in each category for data analysis [67]. This approach allowed us to explore agreement by item and by sub-domain and the opportunity to identify problematic aspects of the training. In the “Technology” sub-domain, it is unknown what technical issues participants encountered and whether they were able to solve them. Future training interventions could explore participants’ connectivity and Internet access prior to the online portion of the Hybrid Course in order to offer alternative ways to access the materials. For instance, if participants’ Internet speed is a challenge to run training videos, the material could be shared in portable storage devices such as USBs, CDs, or DVDs. The comments received in response to the open-ended questions reflected a good acceptance of the training methodology and allowed us to collect suggestions for future training interventions.

### Study limitations

This study has several limitations that are important to note in interpreting the current results and planning for future research studies. While there was a significant increase in post-test scores after the training intervention, it is unknown if improvement in wheelchair service providers’ knowledge translates to better health outcomes in the wheelchair users they served and better competency in wheelchair service provision. Competency is a complex know-how that is based on combining knowledge, skills, abilities and external resources and then applying them appropriately to specific types of situations based on the context [68, 69]. Preliminary studies suggest that the involvement of properly trained professionals contributes to users’ higher satisfaction in the fitting and assignment of assistive technology devices which may turn into improvements in quality of life [70]. Future work should evaluate trainees’ retention of knowledge, improved competency and users’ health outcomes and satisfaction over a long period of time. In this study, we had a small sample size of wheelchair service providers from Colombia, mostly novices, which made our findings not generalizable to wheelchair service providers from other regions of the country and other Latin America settings. Further studies could investigate the impact of the Spanish Hybrid Course across different sub-populations and countries in Latin America to evaluate the effectiveness of this course in building professional capacity in basic level wheelchair service provision.

The Hybrid Satisfaction Survey developed and used in this study was not formally validated. Therefore, we are unsure if the tool measures the underlying outcome of interest [71]. Future work could assess validity and reliability evidence to enhance research quality. Despite this limitation, we used previous satisfaction surveys to guide question development and an international stakeholders’ group to provide further insight, review, and recommendations on the survey.

### Ongoing & future work

Leveraging the potential of online learning has been used as a methodology in LMICs to build capacity in rehabilitation personnel [72]. Other training methodologies such as a complete online course could also be explored, especially to train the trainers for curriculum integration at Universities that offer programs related to wheelchair service provision (e.g., physical therapy, occupational therapy, prosthetics and orthotics, and rehabilitation sciences) [72]. Complementary training such as leadership development for rehabilitation professionals also may be considered in different contexts (e.g., community, facilities, professional organizations, governments) to promote the right to personal mobility of people with disabilities [73].

## Conclusion

Evidence highlights a shortage of education and training in wheelchair service provision in LMICs [21, 22] that results in life complications and secondary health problems related to inappropriate wheelchair provision [14, 21]. The Spanish Hybrid Course proved to be effective in increasing basic level wheelchair knowledge with a high level of satisfaction in a group of wheelchair providers from Colombia. Further research is warranted to evaluate the effectiveness of the Spanish Hybrid Course across different individuals and countries as a tool to build professional capacity in basic level wheelchair provision.

## Supporting information

S1 File Hybrid satisfaction survey–English/Spanish version. (PDF)

S2 File Online and in-person training agenda and learning outcomes. (PDF)

S3 File Dataset. (XLSX)

S4 File Spanish translation of the paper. (PDF)
