# Supplementary material for: Implementation of the hybrid course on basic wheelchair service provision for Colombian wheelchair service providers
Source: PLoS One. 2018 Oct 4;13(10):e0204769. doi: 10.1371/journal.pone.0204769 (PMC6172015; doi:10.1371/journal.pone.0204769)
Supplement: S1 File — (PDF) [file pone.0204769.s001.pdf]

S2. Hybrid Satisfaction Survey - English/Spanish

Hybrid Satisfaction Survey – English version

Sub-domains		Item	0=strongly disagree	1=disagree	2=neither agree nor disagree	3= agree	4= strongly agree
**Interaction**	1	E-learning sessions kept me alert and focused.					
	2	The combination of e-learning sessions and in-person training kept me alert and focused.					
	3	I am satisfied with the quality of interaction between all involved parties (instructor and participants).					
	4	I am satisfied with the way I interacted with other students.					
	5	I am satisfied with my participation in the class.					
**Instructor**	6	In the e-learning format, the instructor(s) was supportive and responsive to my questions.					
	7	In the in-person format, the instructor(s) was supportive and responsive to my questions.					
	8	I was satisfied with the accessibility and availability of the instructor(s).					
	9	I continuously received feedback throughout this course.					
**Instruction methodology**	10	The use of e- learning technology in this course encouraged me to learn independently.					
	11	The use of hands-on activities in this course encouraged me to learn independently.					
	12	After this course, my understanding of wheelchair service provision has improved.					
	13	I am satisfied with the level of effort this course required.					
	14	I am satisfied with my performance in this course.					
	15	I believe I will receive a passing score on the ISWP Basic Test after completing the training.					
	16	I am satisfied with how I will be able to apply what I have learned in this course.					
	17	I am willing to take another course that is part online and part in-person.					
	18	I enjoyed this course.					
	19	I enjoyed working independently in the e-learning portion.					
	20	I enjoyed working collaboratively in the in-person portion.					
**Content**	21	The goals of this course were clearly stated at the beginning of the course.					
	22	My expectations for this course were met.					
	23	In my opinion, the objectives of this course have been accomplished.					
	24	The WHO WSTP Reference Manual supported the learning outcomes for the course.					
	25	Other reading materials assigned were relevant to the course objective.					
	26	Overall, the content of the videos was relevant to the learning outcomes of the course.					
	27	Overall, the online activities were relevant to the learning outcomes of the course.					

	28	Overall, the in-person activities were relevant to the learning outcomes of the course.
	29	I am satisfied with this course and will recommend it to others.
	30	The portion of time I spent doing online activities and time I spent in-person was appropriate
	31	The portion of time I spent doing online activities was appropriate
	32	The portion of time I spent in-person training was appropriate
	33	The material that was selected for the online portion was appropriate.
	34	The material that was selected for the in-person portion was appropriate.
**Technology**	35	Course content shown or displayed on the screen was clear in the e-learning portion.
	36	The video image was clear and comprehensive in the e-learning portion
	37	Overall, the e-learning ran smoothly with few technical difficulties.
	38	Technical problems were not frequent in the e-learning portion.
	39	Technical problems in the e-learning portion do not affect my understanding of the course.
	40	The e-learning methodology is reliable.

Encuesta de Satisfacción del Curso Semipresencial– versión en español

Sub-dominios		Item	0= Totalmente en desacuerdo	1= En desacuerdo	2=Ni de acuerdo ni en desacuerdo	3= De acuerdo	4= Totalmente deacuerdo
**Interacción**	1	Las sesiones de este curso me mantuvieron alerto y concentrado.					
	2	La combinación de sesiones de aprendizaje en línea y sesiones presenciales me mantienen alerto y concentrado.					
	3	Estoy conforme con la calidad de la interacción entre los participantes e instructores de este curso.					
	4	Estoy satisfecho con la forma en la que interactué con otros participantes.					
	5	Estoy satisfecho con mi participación en este curso.					
**Instructor**	6	En las sesiones en línea, los instructores respondieron a mis preguntas y me ofrecieron apoyo.					
	7	En las sesiones presenciales, los instructores respondieron a mis preguntas y me ofrecieron apoyo.					
	8	Estoy conforme con la accesibilidad y disponibilidad de los instructores.					
	9	Recibí retroalimentación a lo largo del curso.					
**Metodología de aprendizaje**	10	Los módulos en línea de este curso me motivaron a aprender de manera independiente.					
	11	Las sesiones presenciales de este curso me motivaron a aprender de manera independiente.					
	12	Después de este curso, mi comprensión del servicio de sillas de ruedas ha mejorado.					
	13	Estoy conforme con el nivel de esfuerzo que este curso requirió.					
	14	Estoy conforme con mi desempeño en este curso.					
	15	Creo que aprobaré el Examen Básico de ISWP una vez finalizado el curso.					
	16	Estoy conforme con cómo aplicaré los conocimientos obtenidos en este curso.					
	17	Estoy dispuesto(a) a tomar otro curso semipresencial.					
	18	Me gustó este curso.					
	19	Disfruté trabajar de forma independiente en la porción en línea.					
	20	Disfruté trabajar de forma colaborativa en la porción presencial.					
**Contenido**	21	Los objetivos de este curso se especificaron con claridad al comienzo del curso.					
	22	El curso cumplió mis expectativas.					
	23	En mi opinión, se cumplieron los objetivos de este curso.					
	24	El manual de referencia de la OMS apoyó el cumplimiento de los objetivos del curso.					
	25	Otros materiales de lectura asignados fueron relevantes para el cumplimiento de los objetivos del curso.					

	26	En general, el contenido de los videos fue relevante para el cumplimiento de los objetivos del curso.
	27	En general, las actividades en línea fueron relevantes para el cumplimiento de los objetivos del curso.
	28	En general, las actividades presenciales fueron relevantes para el cumplimiento de los objetivos del curso.
	29	Estoy satisfecho con este curso y se lo recomendaría a otros.
	30	La cantidad de tiempo que invertí haciendo actividades en línea y la cantidad de tiempo que invertí en actividades presenciales fue adecuada.
	31	La cantidad de tiempo que invertí haciendo actividades en línea fue adecuada.
	32	La cantidad de tiempo que invertí realizando actividades presenciales fue adecuada.
	33	El material que se seleccionó para la parte en línea fue apropiado.
	34	El material que se seleccionó para la parte presencial fue apropiado.
**Tecnología**	35	El contenido del curso mostrado en pantalla durante la porción en línea, fue claro.
	36	La imagen de video, durante la porción en línea, fue clara.
	37	En general, mi aprendizaje en línea transcurrió sin problemas, sólo existieron algunas dificultades técnicas.
	38	Los problemas técnicos no fueron frecuentes en la porción en línea.
	39	Los problemas técnicos en la porción en línea no afectaron mi comprensión del curso.
	40	La metodología de aprendizaje en línea es confiable.
