# Supplementary material for: Implementation of the hybrid course on basic wheelchair service provision for Colombian wheelchair service providers
Source: PLoS One. 2018 Oct 4;13(10):e0204769. doi: 10.1371/journal.pone.0204769 (PMC6172015; doi:10.1371/journal.pone.0204769)
Supplement: S2 File — (PDF) [file pone.0204769.s002.pdf]

S3. Online and in-person training agenda and learning outcomes

Online Modules		
**Time**	**Modules**	**Learning objectives**
**Phase 1**	Introduction	• to describe the hybrid methodology implemented in this course
	A.1 Wheelchair users	• to list at least seven benefits of an appropriate wheelchair for a wheelchair user;<br>• to explain how wheelchair service personnel can support a wheelchair user's right to personal mobility;<br>• to list at least five ways wheelchair users can be actively involved in wheelchair provision.
	A.2 Wheelchair services	• to explain what a wheelchair service is;<br>• to identify their role in a basic wheelchair service;<br>• to define "basic level service" in wheelchair service delivery.
	A.3 Wheelchair mobility	• to introduce basic wheelchair mobility skills safely, including pushing, turning, going up and down slopes, going up and down steps with assistance, and a partial wheelie.
	A.4 Sitting upright	• to list at least 10 features of "sitting upright" (neutral sitting posture);<br>• to list at least six benefits of sitting upright;<br>• to demonstrate four ways that the pelvis can move;<br>• to explain how movement of the pelvis can change sitting posture;<br>• to recognize different sitting postures and how these are different from upright sitting.
**Phase 2**	A.5 Pressure sores	• to list the four stages of a pressure sore;<br>• to recognize when a person with a pressure sore should be referred for specialist help;<br>• to list local services where wheelchair users can be treated for pressure sores;<br>• to list the three main causes of pressure sores;<br>• to identify the main pressure sore problem areas for wheelchair users;<br>• to list risk factors for pressure sores;<br>• to list ways a wheelchair user can prevent pressure sores;
	A.6 Appropriate wheelchair	• to describe at least four things that make a wheelchair "appropriate";<br>• to identify the parts of the wheelchair which affect how the user sits;<br>• to explain how different parts of the wheelchair support specific needs of the wheelchair user;<br>• to identify wheelchairs which are more suited to outdoor/rough terrain than others;<br>• to suggest the most suitable wheelchair to a wheelchair user, considering his/her needs.
	A.7 Cushions	• to list benefits of cushions for wheelchair users;<br>• to name the different types of cushions available locally;<br>• to explain what a "pressure relief cushion" is;<br>• to explain that adding a foam "lift" to a pressure relief cushion can reduce pressure.
	A.8 Transfers	• to list safety points to think about when getting in and out of a wheelchair;<br>• to select at least one transfer method that is best suited for a particular wheelchair user.
**In-person sessions**		
**Time**	**Modules**	**Learning objectives**
**Day 1**	Welcome, introductions and housekeeping	
	A.3 Wheelchair mobility	• to practice basic wheelchair mobility skills covered in the online modules
	A.5 Pressure sores	• to demonstrate two pressure relief techniques.
	A.7 Cushions	• to demonstrate how to check that a pressure relief cushion is reducing pressure;
	A.8 Transfers	• to demonstrate different ways to get in and out of a wheelchair;
	B.1 Referral and appointment	• to describe how wheelchair users may be referred to a wheelchair service;<br>• to describe the appointment system used in the local wheelchair service.
	B.2 Assessment	• to explain the purpose of assessment;<br>• to list the two parts of a wheelchair assessment;<br>• to list information that is gathered during an assessment.

	B.3 Assessment interview	• to demonstrate an assessment interview;<br>• to record information from an assessment interview on a wheelchair assessment form;<br>• to explain how questions in the assessment interview help to choose the most appropriate wheelchair and to identify what a wheelchair user may need to learn.
	B.4 Physical assessment	• to record the presence, risk of or history of pressure sores;<br>• to explain what action should be taken if a wheelchair user has a pressure sore or is at risk of developing a pressure sore;<br>• to identify and record how a wheelchair user will push the wheelchair;<br>• to demonstrate the correct way to take measurements from a wheelchair user for a wheelchair (seat width, seat depth, calf length, back height);<br>• to describe how body measurements relate to wheelchair size.
	B.5 Prescription (selection)	• to list what is included in the prescription (selection) process;<br>• to describe the main features of the wheelchairs that are available locally;<br>• to match wheelchair user needs with the most suitable locally available wheelchair;<br>• to select the correct size of wheelchair for a wheelchair user;<br>• to write down prescription decisions on a wheelchair prescription (selection) form.
**Day 2**	B.6 Funding and ordering	• to review how to order the prescribed wheelchair according to the ordering system of their service.
	Practical 1: Assessment and Prescription	• to demonstrate a basic wheelchair assessment working in a group and with assistance.<br>• to demonstrate making a basic level wheelchair prescription (selection) working in a group and with assistance.
	B.7 Product (wheelchair) preparation	• to adjust a wheelchair to match the wheelchair prescription (selection);<br>• to use the "wheelchair safe and ready" checklist to check that a wheelchair is safe to use and all parts are working.
	B.8 Cushion fabrication	• to point out features of a basic foam pressure relief cushion.<br>• to make a basic foam pressure relief cushion describe how to insert a cushion "lift" for users with unsafe pressure at the seat bones.
	B.9 Fitting	• to list the steps of fitting in the correct order;<br>• to demonstrate how to check the size and common adjustments of a wheelchair for an individual wheelchair user.
	B.10 Problem solving	• to list common wheelchair fit or adjustment problems<br>• to describe simple solutions for common wheelchair fit or adjustment problems.
	B.12 Maintenance and repairs	• to demonstrate how to care for a wheelchair at home;<br>• to identify common technical problems with a wheelchair and explain how these problems can be solved within the local community/context.
**Day 3**	B.11 User training	• to list skills wheelchair service personnel can teach wheelchair users when they receive a new wheelchair;<br>• to list "good practice training methods";<br>• to demonstrate teaching a colleague one wheelchair skill.
	Practical 2: Fitting and user training	• to demonstrate a basic level fitting – with assistance.<br>• to demonstrate providing a wheelchair user with the user training to help him/her to use and maintain the wheelchair effectively.
	Practical 4: Assessment, prescription, fitting and user training	• to demonstrate wheelchair assessment, prescription (selection), product (wheelchair) preparation, fitting and user training at a basic level.
	Practical 1, 2, and 4 Recap	Recap the key points of the three practicals
**Day 4**	B.13 Follow up	• to explain what "follow up" means and how it happens;<br>• to complete a wheelchair's follow up form.
	Practical 3: Follow up	• to demonstrate follow up
	B.14 Putting it all together	• to provide a summary of the wheelchair user they worked with in the last practical session (Practical Four) including: assessment, prescription (selection), product (wheelchair) preparation, fitting, user training and maintenance, repairs and follow up.
