# Supplementary material for: Implementation of the hybrid course on basic wheelchair service provision for Colombian wheelchair service providers
Source: PLoS One. 2018 Oct 4;13(10):e0204769. doi: 10.1371/journal.pone.0204769 (PMC6172015; doi:10.1371/journal.pone.0204769)
Supplement: S4 File — (PDF) [file pone.0204769.s004.pdf]

Implementación del Curso Semipresencial sobre Prestación de Servicios de Sillas de Ruedas Nivel Básico en Proveedores Colombianos

Yohali Burrola-Mendez [1,2,3], Maria Luisa Toro-Hernández [4]; Mary Goldberg [1,2*], Jon Pearlman [1,2]

1. Department of Rehabilitation Science and Technology, University of Pittsburgh, Pittsburgh, Pennsylvania, United States.
2. International Society of Wheelchair Professionals (ISWP), University of Pittsburgh, Pittsburgh, Pennsylvania, United States.
3. Consejo Nacional de Ciencia y Tecnología, México.
4. School of Physical Therapy, Universidad CES, Medellín, Colombia.

Resumen

Introducción: Las personas con movilidad reducida a menudo utilizan sillas de ruedas como su principal medio de movilidad. Los proveedores de servicios de sillas de ruedas no capacitados pueden proporcionar sillas de ruedas y servicios inadecuados que tienen consecuencias negativas en salud, calidad de vida, seguridad y participación social de los usuarios de sillas de ruedas. Este estudio tuvo como objetivo evaluar la influencia del Curso Semipresencial sobre Prestación de Servicios de Sillas de Ruedas Nivel Básico en español, un programa basado en el Paquete de Capacitación en Servicio de Sillas de Ruedas Nivel Básico de la Organización Mundial de la Salud, para aumentar el conocimiento en la prestación de servicios de sillas de ruedas en un grupo de proveedores de servicios de sillas de ruedas de Colombia. Además, desarrollamos una encuesta de satisfacción que los participantes completaron después de la capacitación para comprender los niveles de satisfacción con el Curso Semipresencial en español.

Métodos: Se llevó a cabo un estudio cuasi experimental para evaluar los cambios en el conocimiento del nivel básico de prestación de servicios utilizando la Prueba Básica en Provisión del Servicio de Silla de Ruedas de la Sociedad Internacional de Profesionales de Sillas de Ruedas. Se usaron pruebas *t* de muestras emparejadas para evaluar los cambios en el conocimiento en provisión de servicios sillas de ruedas nivel básico previos y posteriores al entrenamiento. La Encuesta de Satisfacción del Curso Semipresencial se desarrolló en colaboración con un grupo multidisciplinario e internacional de actores interesados. El constructo de interés de la encuesta fue el nivel de satisfacción determinado por la interacción, el instructor, la metodología de instrucción, el contenido y la tecnología, utilizando una escala Likert de cinco puntos (0 = totalmente en desacuerdo a 4 = totalmente de acuerdo). La encuesta se completó de forma anónima después de la intervención educativa y se analizaron los resultados utilizando frecuencias y porcentajes.

Resultados: Quince proveedores de servicios de sillas de ruedas en Colombia completaron el Curso Semipresencial en español. Las puntuaciones medias posteriores al entrenamiento fueron significativamente más altas (media (M)= 56.13, desviación estándar (DE)=7.8), que las puntuaciones previas al entrenamiento (M=50.07, DE=8.38, $t\ (14)=4.923$, $p=<0.0001$). Los participantes que completaron las encuestas (N=15) informaron que el Curso Semipresencial en español fue bien recibido, con 98.66% de las respuestas distribuidas en niveles favorables (>3).

Conclusiones: El Curso Semipresencial en español demostró ser eficaz en el aumento del conocimiento sobre prestación de servicios de la silla de ruedas de nivel básico con una alta satisfacción entre los participantes. Se necesitan más estudios para evaluar la efectividad de este curso en diferentes personas y países como una posible herramienta para desarrollar capacidad profesional en el suministro de sillas de ruedas del nivel básico.

Palabras claves: Educación en línea; Silla de ruedas; Rehabilitación; América Latina; Discapacidad.

Introducción

El Banco Mundial estima que mil millones de personas, o el 15% de la población mundial, experimenta algún tipo de discapacidad y la prevalencia es mayor en los países de ingresos bajos y medianos (PIBM) [1]. En América Latina, el 12,4% de la población, aproximadamente 73 millones de personas, vive con al menos un tipo de discapacidad [2, 3]. La discapacidad es más prevalente entre las mujeres y los grupos económica y socialmente más vulnerables: personas de bajos ingresos, personas de edad avanzada, indígenas, afrodescendientes y habitantes de áreas rurales [2]. De hecho, se estima que alrededor del 80% de las personas con discapacidad en América Latina viven en pobreza, y que las limitaciones en movilidad y visión son los tipos más comunes de discapacidad [2, 4, 5]. La clasificación del Banco Mundial en 2018 sobre el ingreso nacional bruto informa que América Latina está compuesta por 4 países de ingresos mediano-bajo, 12 de ingresos mediano-alto y 4 de ingresos altos [6, 7]. El español es el idioma oficial en el 80% de los países de la región y es el segundo idioma más hablado en el mundo [8, 9]. La Convención de las Naciones Unidas sobre los Derechos de las Personas con Discapacidad (CDPD), que promueve los derechos humanos para todas las personas con discapacidad, ha sido firmada y ratificada por el 95% de los países de América [10-12]. A pesar de los avances en el reconocimiento legal de los derechos de las personas con discapacidad, la situación en América Latina continúa caracterizándose por una gran desigualdad, brechas socioeconómicas y vulnerabilidad social [13].

Las personas con movilidad reducida a menudo utilizan productos de apoyo, como sillas de ruedas, como su principal medio de movilidad. Una silla de ruedas apropiada puede prevenir complicaciones secundarias de salud, mejorar la calidad de vida, la seguridad y facilitar el acceso a otros derechos humanos, como educación, salud y empleo [14-19]. El artículo 20 de la CDPD enfatiza la necesidad de promover la movilidad personal con la mayor independencia al proporcionar capacitación a las personas con discapacidad y al personal que les brinda servicios [20]. Además, los artículos 4, 9, 25, 26 y 32 exigen que el personal capacitado provea sillas de ruedas apropiadas [10]. Sin embargo, la evidencia científica revela que la educación y capacitación en prestación de servicios de sillas de ruedas es insuficiente a nivel mundial [21, 22]. La falta de capacitación puede ocasionar la provisión inadecuada de sillas de ruedas y servicios asociados, lo que provoca efectos negativos en la calidad de vida, la salud, la seguridad y el acceso a otros derechos humanos básicos en las personas con discapacidad [14-20]. Esta situación puede ser más notable en los PIMB donde la incidencia de discapacidad es mayor, existe una gama limitada de sillas de ruedas de calidad asequible, la discapacidad y la pobreza operan en un ciclo, y las personas con discapacidad a menudo son marginadas [2, 21, 23, 24].

La Organización Mundial de la Salud (OMS), con el apoyo de la Agencia de Desarrollo Internacional de los Estados Unidos (USAID, por sus siglas en inglés), publicó una serie de Paquetes de Capacitación en Servicio de Sillas de Ruedas (WSTP de la OMS, por sus siglas en inglés) para ayudar a las naciones a cumplir el mandato de la CDPD de apoyar la capacitación de proveedores y disminuir la brecha en la necesidad de entrenamiento en entornos con menos recursos [14, 25]. Hasta la fecha, hay cinco WSTP de la OMS: nivel básico, nivel intermedio, para administradores, para actores interesados y curso para formación de formadores [25-29]. La OMS ha traducido oficialmente estos paquetes a diferentes idiomas, incluido el español y ofrece acceso gratuito a los materiales del curso desde el sitio web de la OMS [25, 30]. Desde la adopción de la CDPD en 2006, existe un compromiso creciente en América Latina de proporcionar programas educativos y capacitación en provisión de servicios de sillas de ruedas a nivel local para impactar positivamente a la comunidad. Una de esas iniciativas, el Simposio Latinoamericano de Posicionamiento en Sentado y Movilidad, se ha celebrado cada dos años como un

esfuerzo regional para promover el acceso, los estándares de práctica y el uso efectivo de los productos de apoyo [31]. En los simposios, se han impartido talleres de educación continua internacionalmente reconocidos como el Programa de Habilidades en Silla de Ruedas y los WSTP de la OMS [27, 28, 32]; sin embargo, todavía existe una necesidad abrumadora de capacitación y servicios apropiados que no están siendo satisfechos por la fuerza de trabajo actual.

Con el propósito de ofrecer metodologías de aprendizaje alternativas que puedan ser escalables en países y regiones, la Sociedad Internacional de Profesionales de Sillas de Ruedas (ISWP, por sus siglas en inglés) desarrolló en 2016 un Curso Semipresencial en inglés y español utilizando las versiones oficiales del WSTP- nivel básico de la OMS (WHO WSTP-B, por sus siglas en inglés) en ambos idiomas [30, 33]. El Curso Semipresencial consiste en módulos en línea accesibles en entornos con internet de bajo ancho de banda que reducen la exposición de capacitación a 3 días presenciales, lo que reduce el costo y lo hace probablemente escalable [33]. El Curso Semipresencial en inglés demostró ser efectivo en el aumento del conocimiento de provisión de servicios de sillas de ruedas del nivel básico en un grupo de estudiantes de Ciencias de la Rehabilitación en Estados Unidos, con base en los puntajes de la Prueba Básica en Provisión del Servicio de Silla de Ruedas de ISWP [33, 34].

A pesar de que los resultados obtenidos en el Curso Semipresencial en inglés mostraron que esta metodología puede ser efectiva para capacitar sobre provisión de servicios de sillas de ruedas, el curso se probó exclusivamente en inglés, en un entorno con recursos altos y sin obtener información sobre la satisfacción de los participantes [33]. Es necesario evaluar el Curso Semipresencial en español en diferentes contextos y explorar el nivel de satisfacción de los participantes para determinar si es un método de aprendizaje eficaz y bien recibido para capacitar a proveedores de servicios de sillas de ruedas en América Latina. Por lo tanto, el propósito de este estudio exploratorio fue probar el Curso Semipresencial en español en Colombia. Este país fue seleccionado para esta primera intervención educativa debido a la presencia de facilitadores locales que previamente fueron capacitados a través del WSTP- nivel básico de la OMS y el paquete WSTP-curso para formación de formadores de la OMS, organizaciones locales y actores interesados.

Los objetivos específicos de este estudio fueron: (1) desarrollar una encuesta de satisfacción; (2) evaluar el efecto del Curso Semipresencial en español entre un grupo de proveedores de servicios de sillas de ruedas ubicados en Colombia. Nuestra hipótesis fue que los participantes obtendrían puntajes más altos en la Prueba Básica en Provisión del Servicio de Silla de Ruedas de ISWP después de recibir el entrenamiento y que reportarían niveles altos de satisfacción con el entrenamiento. Este estudio proporcionaría una mayor comprensión del Curso Semipresencial en español para ayudar a determinar su potencial implementación en otros países.

Métodos

Cada objetivo específico se completó en secuencia; los métodos se describen a continuación.

Objetivo específico 1: Desarrollo de la encuesta de satisfacción.

Para este objetivo específico, seguimos la metodología implementada en el desarrollo del Curso Semipresencial [33], en el que el mismo grupo internacional y multidisciplinario de actores interesados supervisó y guio el desarrollo de la Encuesta de Satisfacción del Curso Semipresencial. Este grupo, el Subcomité Semipresencial (HSC, por sus siglas en inglés), estaba compuesto por 8 miembros de países de ingresos bajos a altos (Brasil, Canadá, Colombia, India, México, Filipinas, el Reino Unido y los Estados Unidos de América) con experiencia en prestación de servicios de sillas de ruedas y desarrollo de programas de entrenamiento en silla de ruedas [33].

Los autores de este manuscrito y el HSC desarrollaron la Encuesta de Satisfacción del Curso Semipresencial para evaluar los niveles de satisfacción entre los participantes después del entrenamiento utilizando el Curso Semipresencial. El constructo de interés fueron los niveles de satisfacción después de la capacitación determinados por 5 subdominios: interacción, instructor, metodología de instrucción, contenido y tecnología. Los ítems fueron seleccionados de encuestas existentes que evaluaron la satisfacción con el aprendizaje en línea [35], satisfacción con el aprendizaje semipresencial en un entorno con segregación de género [36] y satisfacción con el aprendizaje semipresencial [37]. Los ítems seleccionados se adaptaron para reflejar el contenido del curso. La adaptación de los ítems siguió las mejores prácticas para la construcción de enunciados que incluyeron: usar un lenguaje sencillo, evitar preguntas de doble sentido y escribir enunciados positivos [38, 39]. La encuesta utilizó una escala Likert de cinco puntos (4= totalmente de acuerdo, 3= de acuerdo, 2= ni de acuerdo ni en desacuerdo, 1= en desacuerdo, 0= totalmente en desacuerdo) que indujo a los participantes a indicar el grado en que estaban de acuerdo con cada ítem. Se incluyó una pregunta abierta al final de cada subdominio para alentar a los participantes a proporcionar sugerencias y comentarios. Además, el subdominio "contenido" incluyó una pregunta de opción múltiple que se analizó individualmente: "Para el curso que completó, preferiría: a) tener más módulos en línea, b) tener más práctica en persona, c) tener más material en línea y práctica en persona, d) nada que cambiar, o e) otro (por favor, explique)". La Encuesta de Satisfacción del Curso Semipresencial se completó en línea en Qualtrics®. Dado que la autora principal y 3 miembros de HSC eran bilingües en inglés y español, la Encuesta de Satisfacción del Curso Semipresencial se desarrolló simultáneamente en ambos idiomas y se puede acceder a ella en la sección de Información de Apoyo del artículo (S2. Hybrid Satisfaction Survey).

Objetivo específico 2: Implementar el curso Semipresencial en español con un grupo de proveedores de servicios de sillas en ruedas de Colombia

Diseño del Estudio

El diseño de este estudio es de mediciones previas y posteriores que evaluó los cambios en conocimiento en provisión de servicios de sillas de ruedas nivel básico e incluyó una medición posterior de la satisfacción con el Curso Semipresencial en español en un grupo de proveedores de servicios de sillas de ruedas en Medellín, Colombia. El estudio fue aprobado por el Comité de Ética de la Universidad de Pittsburgh. Se reportaron los resultados del estudio utilizando la lista de verificación TIDieR (Template for Intervention Description and Replication) para el informe de intervenciones [40].

Muestra del estudio

La muestra se seleccionó utilizando un método de muestreo intencional guiado por los actores interesados locales. La Universidad CES en Medellín, Colombia, lideró el reclutamiento, inscripción y facilitación del Curso Semipresencial en español. Los criterios de inclusión incluyeron individuos que: 1) trabajan localmente en la prestación de servicios de sillas de ruedas; 2) que no han tomado la Prueba Básica en Provisión del Servicio de Silla de Ruedas de ISWP. Los participantes que simultáneamente colaboraban en otro estudio relacionado con silla de ruedas fueron excluidos.

La capacitación se proporcionó sin costo para los participantes. Por lo tanto, para reducir la deserción, la Universidad CES envió una convocatoria a los actores interesados en discapacidad y rehabilitación (hospitales, centros de rehabilitación, programas de terapia física, organizaciones no gubernamentales locales e internacionales, líderes comunitarios, entre otros). Las solicitudes de los participantes incluyeron la aprobación de su supervisor y una carta compromiso para completar las actividades de capacitación en línea y presenciales. La Universidad CES seleccionó 15 de las 18 solicitudes recibidas en función de su experiencia laboral y su potencial para difundir el contenido. El número de participantes del curso se determinó en relación a las recomendaciones de la OMS del número de entrenadores por participantes que permiten un entorno de aprendizaje apropiado, ya que el programa cuenta con una cantidad significativa de sesiones prácticas [41].

Intervención

La Tabla 1 presenta la descripción general y la línea de tiempo del estudio y la Tabla 2 proporciona la lista de verificación TIDieR para informar las intervenciones. Tres facilitadoras locales trabajaron de manera interprofesional durante la fase de planificación (reclutamiento de participantes, identificación de usuarios de sillas de ruedas y adquisición de sillas de ruedas) y la facilitación del entrenamiento. Las facilitadoras incluían a una Fisioterapeuta, una Ingeniero Biomédico y una Médico especialista en Medicina Física y Rehabilitación. Las dos primeros participaron en un curso de entrenadores WSTP de la OMS [42]. La Médico tenía más de 10 años de experiencia en el suministro de sillas de ruedas y también recibió capacitación como entrenadora de la Clasificación Internacional de Funcionamiento, Discapacidad y Salud. [43].

Tabla 1. Actividades y línea de tiempo

Actividad	Días	Junio - Septiembre 2016	Octubre 2016				Noviembre 2016			
			1	2	3	4	1	2	3	4
**Reclutamiento**	90									
**Intervención**	42									
Pre-evaluaciones	9									
Capacitación en línea	15									
Fase 1. Módulos 1-*4* *Recitación*	8									
Fase 2. Módulos 5-8 *Recitación*	7									
Capacitación presencial	3.5									
Post-evaluaciones	8									

Tabla 2. Lista de verificación TIDieR

**Nombre de la intervención**	Curso Semipresencial sobre Prestación de Servicios de Sillas de Ruedas Nivel Básico en español de la Sociedad Internacional de Profesionales de Sillas de Ruedas (ISWP)	
**Por qué:**	Es necesario capacitar a los proveedores de servicios de sillas de ruedas de una manera más flexible y escalable. Este programa nunca ha sido probado en español.	
**Qué**	*Materiales*	Adobe Connect, acceso a Internet, un aula grande accesible, camas/colchonetas, sillas de ruedas de demostración, sillas de ruedas para donar, espuma, manuales de referencia los participantes, cuadernos de trabajo de los participantes, manuales de los instructores, formularios de evaluación, carteles, sillas, un pizarrón, computadora, proyector, juego de herramientas para la fabricación de cojines, juego de herramientas para el mantenimiento del hogar. La lista de recursos, materiales y herramientas de capacitación incluidos en el Manual del Instructor del Paquete de Capacitación en Servicio de Sillas de Ruedas – Nivel Básico de la OMS [41].
	Procedimientos	Los participantes tuvieron una semana previa al inicio del curso para completar las pre-evaluaciones. El Curso Semipresencial en español consistió en dos semanas de capacitación asincrónica en línea con dos reuniones en línea sincrónicas (recitaciones). La capacitación en línea consistió en 3.5 días de capacitación presencial. Después de esto, los participantes tuvieron una semana para completar las post-evaluaciones.
**Quién facilitó**	*Capacitación en línea*	Personal de apoyo de ISWP y la autora del Curso Semipresencial en español e inglés.
	Capacitación presencial	Tres instructoras capacitadas (fisioterapeuta, médico e ingeniero biomédico)
**Modelo de entrega**	Un grupo de 15 participantes tomó el Curso Semipresencial en español de forma simultánea y asíncrona. Durante las recitaciones, los participantes interactuaron entre ellos y con los instructores. En las sesiones presenciales, los participantes practicaron con usuarios de sillas de ruedas en grupos de tres.	
**Dónde**	Las sesiones presenciales se facilitaron en un aula de 50 metros cuadrados en la Universidad CES en Medellín, Colombia.	
**Cuando y cuánto**	*Pre-evaluación:*	La Prueba Básica en Provisión del Servicio de Silla de Ruedas de ISWP se completó en línea y toma aproximadamente una hora.
	Capacitación en línea:	Dos semanas, 8 módulos en línea, 2 recitaciones sincrónicas de 90 minutos cada una.
	Capacitación presencial:	Tres días y medio, 8 horas por día.
	Post - evaluaciones:	La Prueba Básica en Provisión del Servicio de Silla de Ruedas de ISWP se completó en línea y toma aproximadamente una hora.<br>La Encuesta de Satisfacción del Curso Semipresencial, se completó en línea y toma aproximadamente 30 minutos.
**Adaptaciones:**	Las sillas de ruedas de demostración utilizadas eran del contexto local.	
**Modificaciones:**	Ninguna	
**Que tan bien**	*Fidelidad:*	No evaluada
	Adherencia:	En general, la intervención se llevó a cabo según lo planeado. Sólo un usuario de silla de ruedas no participó en la capacitación lo que implicó que los participantes tuvieran que trabajar en grupos más grandes. No hay taller de sillas de ruedas en la Universidad CES y se necesitó ajustar una silla de ruedas. El ajuste se realizó en una cita posterior con uno de los instructores y el proveedor de silla de ruedas.

Capacitación en línea

El aprendizaje en línea se dividió en dos fases secuenciales (Tabla 1). En cada fase, los participantes revisaron el contenido y completaron las actividades requeridas de forma asincrónica para cumplir con los objetivos de aprendizaje (S3. Learning Outcomes). La descripción del contenido de los

módulos en línea, las actividades del curso, los materiales de instrucción utilizados y las interacciones de los alumnos se han descrito en otra parte [33]. Después de la finalización de cada fase, se organizó una reunión en línea (recitación) sincrónica entre los participantes y los entrenadores. Durante las recitaciones, los entrenadores reforzaron los puntos clave de los módulos, respondieron preguntas, discutieron temas y promovieron la interacción entre los participantes. Las recitaciones duraron 90 minutos, se grabaron y se pusieron a disposición de los participantes. El personal de ISWP asistió a las recitaciones para ayudar a coordinar las sesiones y brindar apoyo, de ser necesario.

Capacitación presencial

Después de completar la porción en línea del curso, los participantes asistieron a 3.5 días de capacitación presencial dirigida por entrenadores locales en la Universidad CES en Medellín, Colombia (Tabla 1 y S2). Diez usuarios de sillas de ruedas, evaluados previamente por las entrenadoras, fueron invitados a participar como voluntarios en las sesiones presenciales. Además, 5 usuarios de sillas de ruedas que habían recibido una silla de ruedas nueva en el último año fueron invitados a participar como voluntarios en la sesión práctica de seguimiento (S2, Día 4, Práctica 3). El objetivo era que tres participantes trabajaran con un voluntario usuario de silla de ruedas durante cada sesión práctica. El último día, los usuarios de sillas de ruedas recibieron el beneficio de una nueva silla de ruedas apropiada provista por los participantes.

Mediciones de resultados

Se definió como principal medida de interés el cambio de conocimiento y como medida secundaria el nivel de satisfacción de los participantes. Consideramos que éstos dos resultados son relevantes para evaluar la influencia del Curso Semipresencial en español y para planificar futuros estudios.

Para evaluar el cambio de conocimiento, los participantes completaron la Prueba Básica en Provisión del Servicio de Silla de Ruedas de ISWP una semana antes y después del curso. La prueba antes mencionada ha mostrado evidencia científica de su validez para medir el conocimiento de la provisión de servicios de sillas de ruedas de nivel básico independientemente de la ubicación geográfica [34]. La prueba consistió en 19 preguntas sociodemográficas y 75 preguntas de opción múltiple que evaluaron la prestación de servicios básicos de sillas de ruedas. Las preguntas sociodemográficas incluyeron las características generales de los participantes, como edad, sexo, nivel educativo, profesión, situación laboral, años de experiencia en la provisión de servicios de sillas de ruedas, entorno de trabajo y motivación para tomar la capacitación. Algunas preguntas demográficas, como el entorno del trabajo, el grupo de edad servido y la motivación para tomar la capacitación permitieron respuestas múltiples, y se les pidió a los participantes que seleccionaran todas las opciones aplicables. Las 75 preguntas de opción múltiple cubrieron siete dominios de la prestación de servicios de sillas de ruedas: 1) estudio, 2) prescripción, 3) prueba de ajuste, 4) producción, 5) capacitación del usuario, 6) proceso, y 7) seguimiento y mantenimiento como se describe en el WSTP-B de la OMS [34]. Los dominios tenían diferentes ponderaciones según el número de preguntas preestablecido asignado a cada dominio. Cada dominio tenía un conjunto de preguntas creadas para reducir la probabilidad de recibir la misma pregunta al realizar la prueba varias veces. Además, la configuración de prueba incluyó: 1) distribución aleatoria de preguntas y respuestas del conjunto de preguntas de cada dominio; 2) finalización forzada que requirió que los participantes completaran la prueba en una sola entrada; y 3) puntajes de exámenes inmediatos con la capacidad de revisar respuestas correctas e incorrectas [33, 34] Los puntajes de pruebas mayores o iguales a 53 puntos (70% del total de la prueba) se consideraron puntajes de aprobación. Los participantes recibieron un correo electrónico con instrucciones sobre cómo iniciar su

sesión en la plataforma de prueba, Test.com®, e información de contacto del personal de ISWP y el entrenador local en caso de problemas técnicos o preguntas. Los participantes fueron instruidos en completar la prueba sin acceder a los materiales del curso. La prueba fue completada en español. Para evaluar los niveles de satisfacción después de la intervención de capacitación, se invitó a los participantes a completar la Encuesta de Satisfacción del Curso Semipresencial en línea de forma anónima. Los participantes recibieron un correo electrónico con el enlace de la encuesta. La encuesta se realizó en Qualtrics® e incluyó una carta de presentación con instrucciones, el propósito del cuestionario, una declaración de confidencialidad e información de contacto para preguntas o cuestiones técnicas.

Manejo y análisis de datos

Todos los datos fueron recolectados en una base de datos de Test.com® y otra en Qualtrics®, exportados a un archivo CSV y luego a SPSS® Versión 24.0 donde se realizaron todos los análisis. Los datos se inspeccionaron visualmente para evaluar el supuesto de normalidad utilizando gráficos Q-Q. Se calcularon promedios (*M*), desviaciones estándares (*DE*) e intervalos de confianza (IC) del 95%.

Las diferencias entre los puntajes totales y por dominio previos y posteriores de la Prueba Básica en Provisión del Servicio de Silla de Ruedas de ISWP se calcularon y se inspeccionaron visualmente para evaluar el supuesto de normalidad en la distribución de los datos utilizando gráficos Q-Q. Se utilizaron box-plots para presentar gráficamente los puntajes totales y los puntajes de cada dominio previos y posteriores de la Prueba usando porcentajes. Las puntuaciones totales de las pruebas y las puntuaciones de dominio se convirtieron en porcentajes dividiendo la puntuación obtenida por el número total de preguntas (de la prueba o el dominio específico) y luego multiplicando por 100. Los porcentajes se usaron para construir los box-plots de los puntajes del dominio pre y post prueba. Para la primera medida de interés, cambio en el conocimiento, se calculó una prueba *t* de muestras pareadas cuando las diferencias se distribuyeron normalmente. Todos los análisis se llevaron a cabo utilizando un nivel alfa de 0.05.

Para el resultado secundario, la satisfacción de los participantes, las respuestas de la encuesta se analizaron utilizando frecuencias y porcentajes. Cada pregunta se analizó individualmente sumando el número de veces que se seleccionó el tipo de respuesta (4= totalmente de acuerdo, 3= de acuerdo, 2= ni de acuerdo ni en desacuerdo, 1= en desacuerdo, 0= totalmente en desacuerdo). Posteriormente, las frecuencias de cada respuesta se convirtieron en porcentajes dividiendo cada respuesta de la escala tipo Likert entre el número total de respuestas recibidas y luego multiplicando por 100. Las preguntas abiertas se analizaron individualmente.

Resultados

Se reclutó un total de 15 participantes; todos ellos completaron las evaluaciones previas y posteriores y por lo tanto no hubo deserción. Las características de los participantes se describen en la Tabla 3. En cuanto a los entornos de trabajo, la selección más común fue la ambulatoria (n= 9), mientras que la selección menos frecuente fue la de pacientes hospitalizados (n= 3). Referente al "grupo de edad" atendido, todos los participantes seleccionaron "adultos" (n= 15); mientras que "grupo pediátrico" y los "adultos mayores" fueron las segundas opciones más elegidas (n= 9). El "crecimiento profesional" (n= 12) seguido del "crecimiento personal" (n= 7) fueron las motivaciones más comunes para tomar la capacitación.

Tabla 3. Características de los participantes

**Características**	**Curso Semipresencial Colombia (n= 15)**
Edad, media (DE)	36.80 (10.25)
Sexo, Mujer, n (%)	10 (66.7)
Nivel Educativo, n (%)	
<Licenciatura (pregrado)	2 (13.3)
Licenciatura (pregrado)	11 (73.3)
Posgrado	2 (13.3)
Profesión, n (%)	
Terapeuta Ocupacional	1 (6.6)
Fisioterapueta (Terapeuta Físico)	7 (46.6)
Trabajador social	2 (13.3)
Técnico en órtesis y prótesis	1 (6.6)
Ingeniero(a) Biomédico(a)	1 (6.6)
Líder comunitario	1 (6.6)
Educador(ra) Físico	1 (6.6)
Enfermera(o)	1 (6.6)
Situación laboral, n (%)	
20 horas/semana	3 (20.0)
40 horas/semana	12 (80.0)
Entorno de trabajo, n (%)	
Hospital	4 (26.7)
Academia	5 (33.3)
Ambulatorio	9 (60.0)
Pacientes hospitalizados	3 (20.0)
Grupos de edad atendidos, n (%)	
Pediátrico	9 (60.0)
Adolescentes	8 (53.3)
Adultos	15 (100)
Adultos mayores	9 (60.0)

Experiencia provisión de servicios de sillas de ruedas, años, n (%)	
Menos de 3 años	11 (73.3)
De 4-7 años	1 (6.7)
8 o más años	3 (20.0)
Curso previos de sillas de ruedas, n (%)	6 (40)
Tiempo semanal en provisión de sillas de ruedas, horas, n (%)	
Menos de 3 hours	4 (26.7)
De 3-20 horas	8 (53.3)
21 o más horas	3 (20.0)
Motivación para tomar entrenamiento, n (%)	
Crecimiento profesional	12 (80.0)
Crecimiento personal	7 (47.7)
Requerido por programa académico	4 (26.7)
Miembros de alguna organización*, n (%)	11 (73.3)

DE: Desviación Estándar
*Organizaciones que proveen servicios a usuarios de sillas de ruedas

Las diferencias entre las puntuaciones totales previas y posteriores a la Prueba y las puntuaciones previas y posteriores de cada dominio mostraron una distribución normal. La prueba *t* de muestras pareadas indicó que los puntajes posteriores a la evaluación fueron significativamente más altos (M= 56.13, DE= 7.8) a los puntajes de la previos a la capacitación (M= 50.07, DE= 8.38), t(14)=4.923, p=< 0.0001 (Tabla 4).

Tabla 4. Promedio y cambios del promedio en los puntajes totales de la prueba y dominios, N = 15

Prueba (No. Preguntas)	Pre-evaluación N=15			Post-evaluación N=15			Promedio del cambio entre pre/post-evaluaciones (95% CI)
	M (DE)	Min	Max	*M (DE)*	Min	Max	
Dominios							
Estudio (19)	13.93 (2.19)	9	17	16.33 (2.06)	13	19	2.4 (1.38, 3.42) *
Prescripción (12)	7.87 (1.85)	5	11	9.13 (1.92)	5	11	1.26 (-0.04, 2.58)
Prueba de ajuste (10)	4.3 (1.53)	2	7	4.2 (1.78)	0	7	-0.06 (-1.22, 1.08)
Producción (5)	2.8 (1.52)	0	5	3.4 (0.91)	2	5	0.60 (-0.05, 1,25)
Entrenamiento usuario (15)	9.33 (2.29)	5	12	10 (2.3)	4	13	0.66 (-0.86, 2.20)
Proceso (10)	7.13 (2.67)	2	10	8.2 (2.43)	0	10	1.06 (-0.24, 2.38)
Seguimiento y mantenimiento (4)	2.73 (0.703)	2	4	2.87 (1.19)	0	4	0.13 (-0.41, 0.68)
Puntaje total (75)	50.07 (8.38)	34	62	56.13 (± 7.8)	39	68	6.06 (3.42, 8.71)*

M: Media
DE: Desviación estándar
* prueba t de muestras pareadas significativa al nivel p <0.05

La Fig. 1 presenta box-plots de puntuaciones totales previas y posteriores de la Prueba. Se exploró el cambio de conocimiento por dominio; todos los dominios, a excepción del dominio "prueba de ajuste" presentaron un aumento en los puntajes promedio entre la prueba previa y posterior. El dominio "estudio" informó un aumento estadísticamente significativo en los puntajes previos (M= 13.93, DE = 2.19) y los puntajes posteriores de la Prueba (M= 16.33, DE= 2.06), t(14)=5.041, p=<0.0001 (Tabla 4) . En la figura 2, se compararon los box-plots de los siete dominios entre las puntuaciones pre y post prueba.

Se recibieron y analizaron quince Encuesta de Satisfacción del Curso Semipresencial anónimas. Todos los participantes disfrutaron el curso, estuvieron satisfechos con el curso, estuvieron dispuestos a tomar otro curso semipresencial, recomendarían el curso y consideraron que su comprensión sobre el suministro de servicios de sillas de ruedas mejoró. En general, el Curso Semipresencial en español fue evaluado positivamente, informando que 98.66% de las respuestas se distribuyeron en niveles favorables (3= de acuerdo y 4= totalmente de acuerdo) (Fig 3) **(Ver Figuras en documento original).**

Las preguntas abiertas se analizaron individualmente; los resultados mostraron que la mayoría de los estudiantes se sintieron altamente satisfechos con el curso (Tabla 5). Sugerencias para mejorar la porción en línea incluyeron revisar algunas preguntas en el cuestionario y aumentar el tiempo asignado para revisar los módulos A5-A8. Seis participantes sugirieron aumentar el tiempo presencial, 2 sugirieron aumentar tanto el tiempo presencial como el tiempo en línea y 5 sugirieron dejar el curso tal como está.

Tabla 5: Comentarios de los participantes después de la intervención educativa

**Dominios**	**Comentarios**	
	Positivos	Constructivos
**Interacción**	Totalmente satisfecho con el trabajo realizado. Excelentes organizadores e instructores de los temas discutidos. Los temas y su duración fueron apropiados.	
**Instructor**	Todo lo facilitado por los instructores fue excepcional. Estaban preparados para resolver todas las inquietudes y escuchar las propuestas de cada uno de los grupos.	En el curso en línea, hubo algunas preguntas que fueron incorrectas. El tiempo asignado para completar la segunda parte en línea (A5-A8) fue corto.
**Metodología de instrucción**	Nunca había tomado un curso en este formato [semipresencial]. Para ser honesto, tenía miedo. Pero después de tomar este curso, estoy dispuesto a tomar más cursos en este formato.	
**Contenido**	La metodología híbrida es muy interesante porque me permite trabajar individualmente desde mi espacio y en la interacción en persona con profesionales de diferentes campos y experiencias que fortalecen mi trabajo	Me hubiera gustado tener más tiempo en persona para poder ayudar a más usuarios de sillas de ruedas con este curso
**Tecnología**	Solo satisfacción y agradecimiento a los organizadores, patrocinadores, la universidad, los profesores ... ¡excelente!	

Discusión

El presente estudio evaluó la influencia del Curso Semipresencial sobre Prestación de Servicios de Sillas de Ruedas Nivel Básico en español entre un grupo de proveedores de servicios de sillas de ruedas en Medellín, Colombia y desarrollamos una encuesta de satisfacción para capturar el nivel de satisfacción de los participantes después de la capacitación. El Curso Semipresencial en español tuvo una influencia estadísticamente significativa en el puntaje total de la Prueba Básica en Provisión del Servicio de Silla de Ruedas de ISWP y recibió evaluaciones de satisfacción en general altas, demostrando el valor potencial del Curso Semipresencial en español como herramienta para capacitar a proveedores de servicios de sillas de ruedas en el nivel básico.

Este proyecto demuestra una estrategia exitosa que podría abordar la falta de personal capacitado y promover la coordinación entre los profesionales que trabajan con personas con discapacidades. De hecho, la falta de un enfoque multisectorial coordinado, que incluye una desconexión entre diferentes niveles de rehabilitación (por ejemplo, rehabilitación aguda y basada en la comunidad) y una escasez de personal capacitado se han identificado como barreras para lograr una acción global en la agenda de discapacidad en PIBMs [44-47]. Los resultados de este estudio requieren mayor exploración sobre modelos de capacitación escalables y costo-efectivos para construir un sistema de capacitación sostenible, que incluya la capacitación interprofesional, y fomente un enfoque multisectorial coordinado. Por ejemplo, podría explorarse una asociación más sostenible para apoyar el desarrollo de capacidades y una comprensión más profunda de las necesidades sanitarias mundiales, tal como lo proponen los programas de fisioterapia en Irlanda y Uganda [48].

Específicamente para el contexto de Colombia, la Ley de Estatuaria 1618 (Ley de Discapacidad), promulgada en 2013 después de la ratificación de la CDPD, establece que el gobierno garantizará el acceso a las ayudas técnicas (productos de apoyo para las personas con discapacidad) apropiadas para promover la inclusión social, la participación, la habilitación y la rehabilitación integral [49]. Colombia está pasando por una era de post-conflicto que se centra en la construcción de un entorno pacífico mediante la inclusión de grupos vulnerables que tradicionalmente han sido discriminados y marginado [50], incluidas las personas con movilidad reducida que necesitan una silla de ruedas [51]. Sin embargo, el país no cuenta con pautas nacionales para el suministro de sillas de ruedas que incluyan las competencias específicas que los profesionales deben tener para involucrarse en el suministro de sillas de ruedas. Colombia tiene dos programas específicos para el contexto del suministro de sillas de ruedas. Los *Bancos de Ayudas Técnicas* en la capital, Bogotá, que están destinados a proporcionar productos de apoyo, incluyendo sillas de ruedas, a sus residentes más pobres [52]. El requisito para que los profesionales trabajen en provisión de sillas de ruedas es ser un profesional de rehabilitación con capacitación o experiencia en el campo de los productos de apoyo [53]. El otro programa es el *Protocolo Administrativo para la Prescripción, Seguimiento, y Control de los Productos de Apoyo en Salud*, de las fuerzas militares, que requiere una prescripción de un médico y el apoyo de un equipo interdisciplinario de rehabilitación para que los usuarios reciban una silla de ruedas [54]. Ambos programas carecen de detalles sobre las competencias mínimas que los profesionales deben tener para la prestación de servicios de sillas de ruedas. Podría explorarse una intervención según lo propuesto en este estudio para garantizar el conocimiento básico en provisión de servicios de silla de ruedas de los profesionales que trabajan en los programas antes mencionados.

Sesenta y siete por ciento de los puntajes de la prueba preliminar estaban por debajo del límite de aprobación de la prueba (53 puntos), lo que podría indicar la necesidad de actualizar y/o promover la capacitación en la prestación de servicios de sillas de ruedas. Sin embargo, es importante señalar que la mayoría de los participantes (73%) informaron tener menos de tres años de experiencia trabajando con

usuarios de sillas de ruedas. Esta situación puede reflejar el poco tiempo que los programas profesionales otorgan a la capacitación en prestación de servicios de sillas de ruedas [22] y/o la necesidad de integrar éstos temas a los programas curriculares existentes en las universidades para garantizar que los graduados estén adecuadamente preparados y capacitados para asumir el proceso de provisión de sillas de ruedas [21]. Aumentar el tamaño de la muestra e incluir proveedores experimentados en provisión de sillas de ruedas podría ayudar a explorar la posible relación entre el conocimiento básico y la experiencia en la provisión de servicios de sillas de ruedas. El Curso Semipresencial en español tuvo una influencia estadísticamente significativa en el puntaje promedio del grupo en la Prueba Básica en Provisión del Servicio de Silla de Ruedas de ISWP reportando el 74% por ciento de los puntajes posteriores a la prueba por encima del límite de aprobación. Estos hallazgos se correlacionan con nuestro estudio anterior en el que evaluamos el efecto del Curso Semipresencial en inglés en un grupo de estudiantes de Ciencias de Rehabilitación en Estados Unidos [33]. Además, los puntajes promedio se calcularon por dominio para explorar el cambio de conocimiento específico. Todos los dominios, excepto "prueba de ajuste", mostraron un aumento en los puntajes posteriores a la prueba, una observación que también ocurrió en nuestra evaluación previa del Curso Semipresencial en inglés [33]. El "estudio" fue el único dominio que tuvo un aumento estadísticamente significativo. Si bien el objetivo del estudio fue exploratorio y no establecimos como hipótesis cambios estadísticamente significativos en los puntajes totales de las pruebas y los puntajes de dominio, decidimos analizar los resultados mediante la realización de una prueba *t* de muestras pareadas post hoc para recopilar datos para futuros estudios. Es importante señalar que el nivel alfa para las pruebas múltiples no se ajustó porque era un estudio exploratorio con un tamaño de muestra pequeño y nos preocupaba la posibilidad de cometer un error tipo II [55-57]. Aumentar el tamaño de la muestra podría lograrse realizando esta capacitación varias veces en diferentes entornos, cumpliendo con la sugerencia del WSTP de la OMS respecto al tamaño del grupo, para determinar el impacto del Curso Semipresencial en español e inglés en cada dominio específico y detectar modificaciones futuras al contenido, si es necesario.

Una posible fortaleza de este estudio es que actores interesados colaboraron en todas las fases del proceso de investigación. Este enfoque sugiere que los resultados del estudio serán más útiles, relevantes, potencialmente escalables y transferibles a la comunidad [58-60]. Además, el grupo estaba compuesto por diversos profesionales, la colaboración interprofesional ha evidenciado mejores resultados en el cuidado de la salud de las personas lo que indica que debe ser promovida en la capacitación educativa y la atención médica [61]. La literatura específica en el área de provisión de sillas de ruedas indica que se requiere de un equipo interdisciplinario para desarrollar un sistema apropiado que aumente el acceso a la provisión de sillas de ruedas y servicios asociados [14, 21, 25, 62]. Además, las teorías del aprendizaje del adulto indican que las discusiones entre un grupo interprofesional aumentan el conocimiento práctico y la probabilidad de que los individuos dentro del grupo puedan aprender [63, 64]. En esta capacitación, participó un coordinador del programa de productos de apoyo, lo que puede haber ayudado a promover la implementación del programa y abogar por la aceptación del mismo entre otros coordinadores. Es necesario investigar si promover esta capacitación entre coordinadores refleja cambios en la práctica actual. Además, tener un líder comunitario, que también era usuario de una silla de ruedas, se alinea con los Lineamientos Nacionales de Rehabilitación Basada en la Comunidad de Colombia que indican la importancia de contar con trabajadores comunitarios cercanos al sector de la salud para que puedan respaldar las derivaciones, el mantenimiento básico y las reparaciones, así como ayudar al sector a comprender las necesidades reales de las personas con discapacidad y sus familias [65]. Para mejorar el informe de esta intervención de capacitación y en última instancia su replicabilidad, utilizamos la lista de verificación TIDieR, una guía de 12 ítems que mejora la calidad de la descripción de las intervenciones. [40].

Los participantes informaron altos niveles de satisfacción después de la intervención de capacitación en los cinco subdominios de la encuesta de satisfacción: interacción, instructor, metodología de instrucción, contenido y tecnología. Un elemento que pudo influir en la satisfacción de los participantes fue que los facilitadores habían sido capacitados previamente en el curso para formación de formadores,WSTP de la OMS, el cual toma en consideración importantes estrategias de aprendizaje de adultos [42]. Aunado a esto, los facilitadores eran del contexto local, lo que sugiere que están más familiarizados con los problemas contextuales y permiten adaptar el entrenamiento [66]. Se trataron las respuestas tipo Likert como datos ordinales porque no asumimos que la diferencia entre las respuestas era equidistante aunque los números asignados a esas respuestas eran iguales [67]. Como se recomienda en la práctica, utilizamos pruebas no paramétricas que incluyen frecuencias y porcentajes de respuestas en cada categoría para el análisis de datos [67]. Este enfoque nos permitió explorar cada ítem y cada subdominio con la oportunidad de identificar aspectos problemáticos de la capacitación. En el subdominio de "tecnología", se desconoce qué problemas técnicos encontraron los participantes y si fueron capaces de resolverlos. Las futuras intervenciones de capacitación podrían explorar la conectividad de los participantes y el acceso a internet antes de la capacitación en línea del Curso Semipresencial con el fin de ofrecer otras formas de acceder a los materiales. Por ejemplo, si la velocidad de internet de los participantes es un desafío para ejecutar videos de capacitación, el material podría compartirse en dispositivos portátiles de almacenamiento como USB, CD o DVD. Los comentarios recibidos en respuesta a las preguntas abiertas reflejaron una buena aceptación de la metodología de capacitación y nos permitieron recopilar sugerencias para futuras intervenciones de capacitación.

Limitaciones del estudio

Este estudio cuenta con varias limitaciones importantes a considerar en la interpretación de los resultados y la planificación de futuros estudios. Si bien hubo un aumento significativo en los puntajes posteriores a la prueba, no se sabe si la mejora en el conocimiento de los proveedores de servicios de sillas de ruedas se traduce en mejores resultados de salud para los usuarios de sillas de ruedas ni en una mayor competencia en la provisión de servicios de sillas de ruedas. La competencia es un conocimiento complejo que se basa en combinar conocimiento, habilidades, destrezas, recursos externos para aplicarlos de manera apropiada a distintos escenarios del contexto real [68, 69]. Los estudios preliminares sugieren que la participación de profesionales debidamente capacitados está relacionado con una mayor satisfacción de los usuarios de sillas de ruedas lo cual puede significar mejoras en su calidad de vida [70]. Futuros trabajos podrían evaluar la retención de conocimiento de los alumnos, las competencias adquiridas, los aspectos de salud de los usuarios de sillas de ruedas y sus niveles de satisfacción durante un largo período de tiempo. En este estudio, tuvimos un tamaño de muestra pequeño de proveedores de servicios de sillas de ruedas de Colombia, en su mayoría novatos, lo que hizo que nuestros hallazgos no sean generalizables a proveedores de servicios de sillas de ruedas de otras regiones del país y otros entornos de América Latina. Otros estudios podrían investigar el impacto del Curso Semipresencial en español en diferentes subpoblaciones y países de América Latina para evaluar la eficacia de este curso como herramienta para aumentar la capacidad profesional local sobre prestación de servicios de sillas de ruedas de nivel básico.

La Encuesta de Satisfacción del Curso Semipresencial desarrollada y utilizada en este estudio no fue formalmente validada. Por lo tanto, no estamos seguros si la herramienta mide el resultado subyacente de interés [71]. Futuros trabajos podrían evaluar la validez y la fiabilidad de la encuesta para mejorar la calidad de la investigación. A pesar de esta limitación, utilizamos otras encuestas de satisfacción para guiar el desarrollo de las preguntas y un grupo internacional de actores interesados para dar una mayor perspectiva, revisión y recomendaciones sobre la encuesta.

Trabajo en curso y futuro

Aprovechar el potencial del aprendizaje en línea se ha utilizado como una metodología en los PIBMs para desarrollar capacidades en el personal de rehabilitación [72]. También se podrían explorar otras metodologías de capacitación como un curso completo en línea, especialmente para capacitar a los formadores en la integración curricular en Universidades que ofrecen programas relacionados con la prestación de servicios de sillas de ruedas (por ejemplo, terapia física, terapia ocupacional, prótesis y ortopedia y ciencias de la rehabilitación) [72]. La capacitación complementaria, como liderazgo profesional puede ser aplicable en diferentes contextos (por ejemplo, comunidad, instalaciones, organizaciones profesionales, gobiernos) para promover el derecho a la movilidad personal de personas con discapacidad [73].

Conclusión

Existe una limitada educación y acceso a capacitación en la prestación de servicios de sillas de ruedas en PIBM [21, 22] lo que da como resultado problemas de salud secundarios relacionados con el suministro inadecuado de sillas de ruedas [14, 21]. El Curso Semipresencial en español demostró ser eficaz en aumentar el conocimiento sobre la provisión básica de servicios de sillas de ruedas con un alto nivel de satisfacción en un grupo de proveedores de sillas de ruedas de Colombia. Se necesitan investigaciones adicionales para evaluar la efectividad del Curso Semipresencial en español en diferentes personas y países como una herramienta para desarrollar la capacidad profesional en el suministro de sillas de ruedas de nivel básico.

Referencias

1. World Bank. Disability Inclusion 2018 [cited 2018 Jun 05]. Available from: http://www.worldbank.org/en/topic/disability.
2. Economic Commission for Latin America and the Caribbean (ELAC). Social Panorama of Latin America. Briefing paper. 2012.
3. Worldometers. Latin America and the Caribbean Population 2018 [cited 2018 May 08]. Available from: http://www.worldometers.info/world-population/latin-america-and-the-caribbean-population/.
4. World Bank. World Development Indicators: Population 2016 [cited 2018 April 17]. Available from: http://databank.worldbank.org/data/reports.aspx?source=2&country=LCN.
5. World Bank. Disability in Latin America and the Caribbean - Fact sheet [cited 2018 April 23,]. Available from: http://siteresources.worldbank.org/DISABILITY/Resources/Regions/LAC/LACfactsheetEng.pdf.
6. World Bank. The World Bank In Latin America and the Caribbean 2018 [cited 2018 April 20]. Available from: http://www.worldbank.org/en/region/lac/overview.
7. World Bank. World Bank Country and Leading Groups: Country Classification 2018 [cited 2018 April 20]. Available from: https://datahelpdesk.worldbank.org/knowledgebase/articles/906519-world-bank-country-and-lending-groups.
8. Simons G, Fenning Ce. Ethnologue: Languages of the World 2018 [cited 2018 April 17,]. Available from: https://www.ethnologue.com/browse/countries - quicktabs-browse_the_countries_of_the_worl=2.
9. World Economic Forum. These are the world's most spoken languages 2018 [cited 2018 August, 01]. Available from: https://www.weforum.org/agenda/2018/02/chart-of-the-day-these-are-the-world-s-most-spoken-languages/.
10. United Nations, Division for Social Policy and Development Disability. Convention on the Rights of Persons with Disabilities (CRPD) New York: United Nations; 2006 [cited 2018 Jan 3]. Available from: https://www.un.org/development/desa/disabilities/convention-on-the-rights-of-persons-with-disabilities.html.
11. Worldometers. Countries in Latin America and the Caribbean: Worldometers. Available from: http://www.worldometers.info/geography/how-many-countries-in-latin-america/.
12. United Nations Treaty Collection. Convention on the Rights of Persons with Disabilities (CRPD): United Nations; 2018 [updated April 17, 2018; cited 2018 April 17]. Available from: https://treaties.un.org/Pages/ViewDetails.aspx?src=TREATY&mtdsg_no=IV-15&chapter=4&clang=_en.
13. Stang-Alva MF. Las personas con discapacidad en América Latina: del reconocimiento jurídico a la desigualdad real. Santiago de Chile: Comisión Económica para América Latina y el Caribe (CEPAL), 2011.
14. World Health Organization. Guidelines on the provision of manual wheelchairs in less resourced settings. Geneva: WHO; 2008.
15. United Nations. The Universal Declaration of Human Rights [cited 2018 April 20]. Available from: http://www.un.org/en/universal-declaration-human-rights/.
16. Toro ML, Eke C, Pearlman J. The impact of the World Health Organization 8-steps in wheelchair service provision in wheelchair users in a less resourced setting: a cohort study in Indonesia. BMC Health Serv Res. 2016;16:26. Epub 2016/01/24. doi: 10.1186/s12913-016-1268-y. PubMed PMID: 26801984; PubMed Central PMCID: PMCPMC4722611.

17. Borg J, Lindstrom A, Larsson S. Assistive technology in developing countries: national and international responsibilities to implement the Convention on the Rights of Persons with Disabilities. Lancet. 2009;374(9704):1863-5. Epub 2009/12/01. doi: 10.1016/S0140-6736(09)61872-9. PubMed PMID: 19944867.
18. Visagie S, Scheffler E, Schneider M. Policy implementation in wheelchair service delivery in a rural South African setting. Afr J Disabil. 2013;2(1):63. Epub 2013/09/09. doi: 10.4102/ajod.v2i1.63. PubMed PMID: 28729993; PubMed Central PMCID: PMCPMC5442587.
19. Carver J, Ganus A, Ivey JM, Plummer T, Eubank A. The impact of mobility assistive technology devices on participation for individuals with disabilities. Disabil Rehabil Assist Technol. 2016;11(6):468-77. Epub 2015/03/31. doi: 10.3109/17483107.2015.1027295. PubMed PMID: 25815679.
20. United Nations, Division for Social Policy and Development Disability. Convention on the Rights of Persons with Disabilities – Articles. Geneva: United Nations; 2006 [cited 2018 Jan 3]. Available from: https://www.un.org/development/desa/disabilities/convention-on-the-rights-of-persons-with-disabilities/convention-on-the-rights-of-persons-with-disabilities-2.html.
21. McSweeney E, Gowran RJ. Wheelchair service provision education and training in low and lower middle income countries: a scoping review. Disabil Rehabil Assist Technol. 2017:1-13. Epub 2017/11/03. doi: 10.1080/17483107.2017.1392621. PubMed PMID: 29092684.
22. Fung KH, Rushton PW, Gartz R, Goldberg M, Toro ML, Seymour N, et al. Wheelchair service provision education in academia. Afr J Disabil. 2017;6:340. Epub 2017/09/25. doi: 10.4102/ajod.v6i0.340. PubMed PMID: 28936415; PubMed Central PMCID: PMCPMC5594266 may have inappropriately influenced them in writing this article.
23. World Health Organization. World Report on Disability. Geneva: 2011.
24. Banks LM, Kuper H, Polack S. Poverty and disability in low- and middle-income countries: A systematic review. PLoS One. 2017;12(12):e0189996. Epub 2017/12/22. doi: 10.1371/journal.pone.0189996. PubMed PMID: 29267388; PubMed Central PMCID: PMCPMC5739437.
25. World Health Organization. Wheelchair Service Training Package: Basic Level. Geneva: WHO; 2012.
26. World Health Organization. Wheelchair Service Training Package: Intermediate Level. Geneva: WHO; 2013.
27. World Health Organization. Wheelchair Service Training Package: Managers. Geneva: WHO; 2015.
28. World Health Organization. Wheelchair Service Training Package: Stakeholders Geneva: WHO; 2015.
29. World Health Organization. Wheelchair Service Training of Trainers Package. Geneva: WHO; 2018.
30. Organización Mundial de la Salud. Paquete de Capacitación en Servicicio de Silla de Ruedas: Nivel Básico. Geneva: OMS; 2014.
31. IV Simposio Latinoamericano de Posicionamiento en Sentado y Movilidad [cited 2018 May 14]. Available from: http://www.seating.com.ar/es/quienes_somos.php.
32. Dalhousie University. Wheelchair Skills Program 2012 [Mar 01 2018]. Available from: http://www.wheelchairskillsprogram.ca/eng/index.php.
33. Burrola-Mendez Y, Goldberg M, Gartz R, Pearlman J. Development of a Hybrid Course on Wheelchair Service Provision for clinicians in international contexts. PLoS One. 2018;13(6):e0199251.

Epub 2018/06/16. doi: 10.1371/journal.pone.0199251. PubMed PMID: 29906794; PubMed Central PMCID: PMCPMC6003808.
34. Gartz R, Goldberg M, Miles A, Cooper R, Pearlman J, Schmeler M, et al. Development of a contextually appropriate, reliable and valid basic Wheelchair Service Provision Test. Disabil Rehabil Assist Technol. 2016;12(4):333-40. Epub 2016/04/22. doi: 10.3109/17483107.2016.1166527. PubMed PMID: 27100362.
35. Roach V, Lemasters L. Satisfaction with Online Learning: A Comparative Descriptive Study Journal of Interactive Online Learning. 2006;5(3).
36. Naaj M, Nachouki M, Ankit A. Evaluating student satisfaction with blended learning in a gender-segregated environment. Journal of Information Technology Education: Research. 2012;11:185-200.
37. Al-Hassan S, Shukri N. The Effect of Blended Learning in Enhancing Female Students' Satisfaction in the Saudi Context. English Language Teaching. 2017;10(6):190-203.
38. Nemoto T, Beglar D, editors. Developing Likert-scale questionnaires. JALT2013; 2014; Tokio:JALT.
39. Tait A, Voepel-Lewis T. Survey research: it's just a few questions, right? Pediatric Anesthesia. 2015;25(1):656-62.
40. Hoffmann TC, Glasziou PP, Boutron I, Milne R, Perera R, Moher D, et al. Better reporting of interventions: template for intervention description and replication (TIDieR) checklist and guide. BMJ. 2014;348:g1687. Epub 2014/03/13. doi: 10.1136/bmj.g1687. PubMed PMID: 24609605.
41. World Health Organization. Wheelchair Service Training Package. Trainer's Manual Basic Level. Geneva: WHO; 2012. 248 p.
42. Munera S, Goldberg M, Kandavel K, Pearlman J. Development and evaluation of a wheelchair service provision training of trainers programme. Afr J Disabil. 2017;6:360. Epub 2017/09/25. doi: 10.4102/ajod.v6i0.360. PubMed PMID: 28936423; PubMed Central PMCID: PMCPMC5594273.
43. International Classification of Functioning DaH. ICF Workshops 2017 [cited 2018 May 09]. Available from: https://www.icf-research-branch.org/icf-training/icf-workshops.
44. Khan F, Amatya B, Mannan H, Burkle FM, Jr., Galea MP. Rehabilitation in Madagascar: Challenges in implementing the World Health Organization Disability Action Plan. J Rehabil Med. 2015;47(8):688-96. Epub 2015/07/02. doi: 10.2340/16501977-1995. PubMed PMID: 26132505.
45. Khan F, Amatya B, Sayed TM, Butt AW, Jamil K, Iqbal W, et al. World Health Organisation Global Disability Action Plan 2014-2021: Challenges and perspectives for physical medicine and rehabilitation in Pakistan. J Rehabil Med. 2017;49(1):10-21. Epub 2017/01/20. doi: 10.2340/16501977-2149. PubMed PMID: 28101563.
46. Khan F, Amatya B, Avirmed B, Yi YK, Shirmen B, Abbott G, et al. World Health Organization Global Disability Action Plan: The Mongolian Perspective. J Rehabil Med. 2018;50(4):388-66. Epub 2017/02/22. doi: 10.2340/16501977-2207. PubMed PMID: 28218340.
47. World Health Organization. WHO Global Disability Action Plan 2014-2021: Better health for all people with disability. 2015.
48. O'Sullivan C, Kazibwe H, Whitehouse Z, Blake C. Constructing a Global Learning Partnership in Physiotherapy: An Ireland-Uganda Initiative. Front Public Health. 2017;5:107. Epub 2017/07/01. doi: 10.3389/fpubh.2017.00107. PubMed PMID: 28660180; PubMed Central PMCID: PMCPMC5466950.
49. El Congreso de Colombia. Ley Estatuaria 1618 de 2013 [cited 2018 May 12]. Available from: https://discapacidadcolombia.com/phocadownloadpap/LEGISLACION/LEY ESTATUTARIA 1618 DE 2013.pdf.

50. International Association for Humanitarian Policy and Conflict Research. Empowerment: Persons with Disabilities Peace building initiative [cited 2018 May 12]. Available from: http://www.peacebuildinginitiative.org/indexd147.html?pageId=1964.
51. Acuerdo final para la terminación del conflicto y la construcción de una paz estable y duradera: Oficina de Alto Comisionado para la Paz; 2016. Available from: http://www.altocomisionadoparalapaz.gov.co/procesos-y-conversaciones/Documentos compartidos/24-11-2016NuevoAcuerdoFinal.pdf.
52. Rincón AR, Gutiérrez RL, Olivera RM, MC V. Lineamientos para el otorgamiento de ayudas técnicas. 2007:107.
53. Camargo DA, Lancheros EY, Peñaloza IS, Mora F, Amórtegui DA. Proceso de actualización de las guías del Banco de Ayudas Técnicas 2011 [cited 2018 May 12]. Available from: http://salud.univalle.edu.co/escuelas/rehabilitacion/Logros/Publicaciones/522_proceso_de_actualizacion.pdf.
54. Protocolo Administrativo para la prescripción, seguimiento y control de los Productos de Apoyo en Salud (PAS) para el SSFM DGSM, (11/11/2015, 2015).
55. Jaeger R, Halliday T. On confirmatory versus exploratory research. Herpetologica. 1998:S64-S6.
56. Moore C, Carter R, Nietert P, Stewart P. Recommendations for Planning Pilot Studies in Clinical and Translational Research. Clinical and translational science. 2011;4(5):332-7.
57. Perneger TV. What's wrong with Bonferroni adjustments. BMJ. 1998;316(7139):1236-8. Epub 1998/05/16. PubMed PMID: 9553006; PubMed Central PMCID: PMCPMC1112991.
58. Camden C, Shikako-Thomas K, Nguyen T, Graham E, Thomas A, Sprung J, et al. Engaging stakeholders in rehabilitation research: a scoping review of strategies used in partnerships and evaluation of impacts. Disabil Rehabil. 2015;37(15):1390-400. Epub 2014/09/23. doi: 10.3109/09638288.2014.963705. PubMed PMID: 25243763.
59. Bowen SJ, Graham ID. From knowledge translation to engaged scholarship: promoting research relevance and utilization. Arch Phys Med Rehabil. 2013;94(1 Suppl):S3-8. Epub 2012/11/13. doi: 10.1016/j.apmr.2012.04.037. PubMed PMID: 23141502.
60. Hinchcliff R, Greenfield D, J B. Is it worth engaging in multi-stakeholder health services research collaborations? Reflections on key benefits, challenges and enabling mechanisms. International journal for quality in health care 2014;26(2):124-8.
61. Mahler C, Gutmann T, Karstens S, Joos S. Terminology for interprofessional collaboration: definition and current practice. GMS Z Med Ausbild. 2014;31(4):Doc40. Epub 2014/12/10. doi: 10.3205/zma000932. PubMed PMID: 25489340; PubMed Central PMCID: PMCPMC4259059.
62. Sheldon S, NA J. ISPO consensus conference on wheelchairs for developing countries: Conclusions and recommendations. Prosthetics and Orthotics International. 2007;31(2):217-23.
63. Taylor DC, Hamdy H. Adult learning theories: implications for learning and teaching in medical education: AMEE Guide No. 83. Med Teach. 2013;35(11):e1561-72. Epub 2013/09/06. doi: 10.3109/0142159X.2013.828153. PubMed PMID: 24004029.
64. Luft J, Ingham H, editors. The JoHari window, a graphic model of interpersonal awareness. Proceedings of the western training laboratory in group development; 1955; Los Angeles, CA: UCLA.
65. Grupo de Gestión en Discapacidad Oficina de Promoción Social. Lineamientos Nacionales de Rehabilitación Basada en la Comunidad (RBC) Ministerio de Salud y Protección Social2014 [cited 2018 May 13]. Available from: https://discapacidadcolombia.com/phocadownloadpap/GOBIERNO/lineamientos-nacionales-rbc.pdf.
66. Yarber L, Brownson CA, Jacob RR, Baker EA, Jones E, Baumann C, et al. Evaluating a train-the-trainer approach for improving capacity for evidence-based decision making in public health. BMC

Health Serv Res. 2015;15:547. Epub 2015/12/15. doi: 10.1186/s12913-015-1224-2. PubMed PMID: 26652172; PubMed Central PMCID: PMCPMC4676893.
67. Sullivan GM, Artino JA. Analyzing and interpreting data from Likert-type scales. Journal of graduate medical education. 2013;5(4):541-2.
68. Fullerton JT, Thompson JB, Johnson P. Competency-based education: the essential basis of pre-service education for the professional midwifery workforce. Midwifery. 2013;29(10):1129-36. Epub 2013/08/03. doi: 10.1016/j.midw.2013.07.006. PubMed PMID: 23906879.
69. Goudreau J, Pepin J, Dubois S, Boyer L, Larue C, Legault A. A second generation of the competency-based approach to nursing education. Int J Nurs Educ Scholarsh. 2009;6:Article15. Epub 2009/05/05. doi: 10.2202/1548-923X.1685. PubMed PMID: 19409071.
70. Schiappa V, Lee-Houser C, Schmeler M, Schein R, Pramana G, Saptono A, editors. Impact of the Assistive Technology Professional (ATP) in the Provision of Mobility Assistive Equipment. The 34th International Seating Symposium; 2018; Vancouver, BC Canada.
71. Sullivan GM. A primer on the validity of assessment instruments. J Grad Med Educ. 2011;3(2):119-20. Epub 2012/06/02. doi: 10.4300/JGME-D-11-00075.1. PubMed PMID: 22655129; PubMed Central PMCID: PMCPMC3184912.
72. O'Brien L, Broom L, Ullah MM. Outcomes and participant experience of an online train-the-trainer program for Bangladeshi health professionals: a case study evaluation. J Contin Educ Health Prof. 2015;35(1):46-56. Epub 2015/03/25. doi: 10.1002/chp.21262. PubMed PMID: 25799972.
73. Pascal MR, Mann M, Dunleavy K, Chevan J, Kirenga L, Nuhu A. Leadership Development of Rehabilitation Professionals in a Low-Resource Country: A Transformational Leadership, Project-Based Model. Front Public Health. 2017;5:143. Epub 2017/07/12. doi: 10.3389/fpubh.2017.00143. PubMed PMID: 28691003; PubMed Central PMCID: PMCPMC5481310.
